# Functional Characterization and Evaluation of *In Vitro* Protective Efficacy of Murine Monoclonal Antibodies BURK24 and BURK37 against *Burkholderia pseudomallei*

**DOI:** 10.1371/journal.pone.0090930

**Published:** 2014-03-10

**Authors:** Bhavani V. Peddayelachagiri, Soumya Paul, Shivakiran S. Makam, Radhika M. Urs, Joseph J. Kingston, Urmil Tuteja, Murali H. Sripathy, Harsh V. Batra

**Affiliations:** 1 Microbiology Division, Defence Food Research Laboratory, Mysore, Karnataka, India; 2 Microbiology Division, Defence Research and Development Establishment, Gwalior, Madhya Pradesh, India; Wadsworth Center, New York State Dept. Health, United States of America

## Abstract

*Burkholderia pseudomallei*, the causative agent of melioidosis has been recognized by CDC as a category B select agent. Although substantial efforts have been made for development of vaccine molecules against the pathogen, significant hurdles still remain. With no licensed vaccines available and high relapse rate of the disease, there is a pressing need for development of alternate protection strategies. Antibody-mediated passive protection is promising in this regard and our primary interest was to unravel this frontier of specific mAbs against *Burkholderia pseudomallei* infections, as functional characterization of antibodies is a pre-requisite to demonstrate them as protective molecules. To achieve this, we designed our study on *in vitro*-based approach and assessed two mAbs, namely BURK24 and BURK37, reactive with outer membrane proteins and lipopolysaccharide of the pathogen respectively, for their ability to manifest inhibitory effects on the pathogenesis mechanisms of *B. pseudomallei* including biofilm formation, invasion and induction of apoptosis. The experiments were performed using *B. pseudomallei* standard strain NCTC 10274 and a clinical isolate, *B. pseudomallei* 621 recovered from a septicemia patient with diabetic ailment. The growth kinetic studies of the pathogen in presence of various concentrations of each individual mAb revealed their anti-bacterial properties. Minimal inhibitory concentration and minimal bactericidal concentration of both the mAbs were determined by using standards of Clinical and Laboratory Standards Institute (CLSI) and experiments were performed using individual mAbs at their respective bacteriostatic concentration. As an outcome, both mAbs exhibited significant anti-*Burkholderia pseudomallei* properties. They limited the formation of biofilm by the bacterium and completely crippled its invasion into human alveolar adenocarcinoma epithelial cells. Also, the mAbs were appreciably successful in preventing the bacterium to induce apoptosis in A549 cells. The present study design revealed the protection attributes possessed by BURK24 and BURK37 that has to be further substantiated by additional *in vivo* studies.

## Introduction

*Burkholderia pseudomallei* infections, collectively termed as melioidosis, are endemic in Southeast Asia and northern Australia. The disease is fatal with a mortality rate of about 40% among treated patients [1]. Clinical manifestations in case of melioidosis vary from acute febrile illness, chronic localized pathologic symptoms to chronic septicaemia resulting in septic shock [1], [2]. In Thailand, *B. pseudomallei* infections are predominantly manifested as community-acquired septicaemia. The infection recommends longer course of intensive antibiotic therapy that comprises of intravenous administration of ceftazidime for 10 days, followed by oral antimicrobial therapy comprising of trimethoprim and sulfamethoxazole for 3–6 months [3]. However, despite of adequate antibiotic therapy the pathogen is capable of undergoing latency for decades and relapse in later stages of immune suppressed conditions [4]. Furthermore, intrinsic resistance of *B. pseudomallei* to multiple drugs and emergence of resistant strains against the above mentioned antibiotic regimen has become a challenge for curing melioidosis [5], [6], [7], [8]. No substitutional protecting molecules or human vaccines for the disease currently exist. Therefore, there is a high priority for development of alternative antimicrobial molecules that can efficiently confer passive protection against the disease among the susceptible hosts. Also, generation of immediate protection in infected hosts is required in order to overcome *B. pseudomallei* infections.

To combat the aforementioned challenge, passive antibody-mediated protection would be a better alternative as antibodies upon passive administration confer immediate and specific immunity to the susceptible and/or infected host. Additionally, antibodies have minimal toxicity as they are natural in origin. Monoclonal antibodies (mAbs) hold great promise in this view, since the antimicrobial activity conferred by them shall be highly specific. Thus, they are also growing faster as new class of therapeutic and passive protection molecules [9], [10].

Pathogenesis mechanisms of *B. pseudomallei* are being explored intensively both *in vitro* and *in vivo*. As a pathogen, it has several strategies to survive and establish pathogenesis in host system. The key factor of its pathogenesis is the ability to survive intracellularly in both phagocytic and non-phagocytic cells [11]. This feature makes the pathogen successful in escaping host immune responses, establish chronic infections, latency and multi-drug resistance. Once invaded into host cells, *B. pseudomallei* induces cell-to-cell fusion resulting in formation of multinucleated giant cells (MNGC) [12]. This is followed by the induction of apoptosis in infected cells by unknown mechanism [13]. The inherent ability of the pathogen to form biofilm is also likely to be responsible for persistence of infection [14]. *B. pseudomallei* has demonstrated resistance to antimicrobial drugs when grown in conditions that induce biofilm formation [15]. Development of specific antibodies against the bacterial components involved in adherence and invasion into host cells might possibly prevent the pathogen to establish pathogenesis and in turn confer passive protection in susceptible and infected hosts. Earlier works reported on similar lines implied the promise of structural virulent factors present on the surface of bacteria for the purpose, since location of the antigens plays an important role in determining the effectiveness of the antibodies [16], [17], [18]. Further, the antibodies, when bound to the surface-expressed antigens may enhance phagocytosis [19] or trigger complement fixation resulting in subsequent killing of internalized bacteria [20]. Targeting a virulence factor that plays a key role in the former mentioned pathogen-mechanisms appears to be promising.

However, according to a recent review, passive immunization against *B. pseudomallei* using mAbs raised against surface expressed molecules including capsular polysaccharide (CPS), lipopolysaccharide (LPS) and exopolysaccharide provide significant but not complete protection in murine models [21]. Also, using mAbs raised against CPS and LPS of *B. pseudomallei* in different studies have shown partial protection in murine models [22], [23]. The mAb-based passive immunization studies reported earlier failed to confer complete infection sterilization [24] and protection against different challenge routes [25]. Prior to *in vivo* challenge studies, characterization of mAbs for their functional ability to block the pathogen mechanisms and also elucidation of the mode of protection they confer to the host is considerable in selection of promising monoclonal antibody to fight against *B. pseudomallei* infections.

With this background, we attempted to develop mAbs possessing protective properties against *B. pseudomallei* infections. Here, our principal goal was to systematically investigate the “functional properties” of specific monoclonal antibodies in conferring protection against *B. pseudomallei* infections. An extended proof through *in vitro*-based experimental models was generated to study the effectiveness of mAbs in mounting reasonable anti-*Burkholderia pseudomallei* activities. Accordingly, two mAbs, namely BURK24 and BURK37, were generated against the crude outer membrane proteins (OMPs) extract of *B. pseudomallei* and exploited in the present study. A novel indirect immunofluorescence assay was developed to study the binding of mAbs on the bacterial surface. Both the mAbs were studied for their kinetics in bacteriostasis and bacterial killing. *In vitro* based biofilm and invasion inhibition assays were also performed to examine the anti-*Burkholderia pseudomallei* properties of BURK24 and BURK37. The mAbs were evaluated for their ability to prevent pathogen-induced apoptosis as well.

## Materials and Methods

### Ethics Statement

BALB/c mice used in this study were obtained from Central Animal Facility, Defence Food Research Laboratory (DFRL), and housed under pathogen free condition. All the animal experiments were performed with the approval and written consent of the Animal Welfare and Research Ethics Committee of DFRL.

### Bacterial Strains and Media

A standard strain of *Burkholderia pseudomallei* NCTC 10274 used in this study was received from Defence Research and Development Establishment, Gwalior, India. A clinical isolate of the bacterium, isolated from a case of melioidosis in a diabetic patient [26] was obtained from Dr. RD Kulakarni, Head, Dept. of Microbiology, SDM Medical College and Hospital, Dharwad, India. The mentioned clinical isolate is referred as *B. pseudomallei* 621 in the present study. Rest of the *Burkholderia* isolates used in this study were isolated from environmental samples including soil and water using Ashdown's selective medium, as described previously [27]. All other bacteria listed in Table 1 were the standard strains obtained from ATCC (USA), MTCC (Chandigarh, India) and NCIM (Imtech, Pune, India). Cultures were maintained in trypticase soy broth (TSB), Luria Bertani (LB) broth and Mueller Hinton broth (MHB) and Mueller Hinton agar (MHA) (Himedia, India); adjusted to 0.5 McFarland standard when required. Antibiotics were used at the final concentration of 4 µg/ml for gentamicin and 100 µg/ml for ampicillin.

**Table 1:** Bacterial strains used in this study and their reactivity with the stabilized monoclonal antibodies.

Sl. No.	Bacteria	BURK21	BURK23	BURK24	BURK33	BURK34	BURK36	BURK37	BURK38	BURK39
1	*Burkholderia pseudomallei*	1/11	1/11	11/11	1/11	1/11	1/11	11/11	8/11	1/11
2	*Burkholderia mallei* *NCTC 10674	0/1	0/1	0/1	0/1	0/1	0/1	0/1	0/1	0/1
3	*Burkholderia cepacia* **MTCC 1617	0/1	1/1	1/1	1/1	0/1	1/1	1/1	0/1	0/1
4	*Burkholderia gladioli* MTCC 1888	0/1	0/1	0/1	0/1	0/1	0/1	0/1	0/1	0/1
5	*Pseudomonas aeruginosa*	0/2	1/1	0/2	0/2	0/2	0/2	0/2	0/2	0/2
6	*Pseudomonas taetrolens* MTCC 1612	0/1	1/1	0/1	0/1	0/1	0/1	0/1	0/1	0/1
7	*Pseudomonas putida* MTCC 102	0/1	1/1	0/1	0/1	0/1	0/1	0/1	0/1	0/1
8	*Pseudomonas alcaligenes* MTCC 493	0/1	1/1	0/1	0/1	0/1	0/1	0/1	0/1	0/1
9	*Pseudomonas citronellolis* MTCC 1191	0/1	1/1	0/1	0/1	0/1	0/1	0/1	0/1	0/1
10	*Pseudomonas mucidolens* MTCC 1618	0/1	1/1	0/1	0/1	0/1	0/1	0/1	0/1	0/1
11	*Pseudomonas synxantha* MTCC 2652	0/1	1/1	0/1	0/1	0/1	0/1	0/1	0/1	0/1
12	*Pseudomonas syringae* MTCC 1604	0/1	1/1	0/1	0/1	0/1	0/1	0/1	0/1	0/1
13	*Pseudomonas* sp. MTCC 512	0/1	1/1	0/1	0/1	0/1	0/1	0/1	0/1	0/1
14	*Pseudomonas fragi* MTCC 510	0/1	1/1	0/1	0/1	0/1	0/1	0/1	0/1	0/1
15	*Pseudomonas azotogensis* ***NCIM 2075	0/1	1/1	0/1	0/1	0/1	0/1	0/1	0/1	0/1
16	*Ralstonia eutropha* MTCC 1954	0/1	0/1	0/1	0/1	0/1	0/1	0/1	0/1	0/1
17	*Brevundimonas diminuta* MTCC 1287	0/1	0/1	0/1	0/1	0/1	0/1	0/1	0/1	0/1
18	*Acidovorax facilis* MTCC 1198	0/1	0/1	0/1	0/1	0/1	0/1	0/1	0/1	0/1
19	*Aeromonas hydrophila*	0/3	3/3	0/3	0/3	0/3	0/3	0/3	0/3	0/3
20	*Aeromonas sobria* isolate	0/1	1/1	0/1	0/1	0/1	0/1	0/1	0/1	0/1
21	*Plesiomonas shigelloides* NCIM 1737	0/1	1/1	0/1	0/1	0/1	0/1	0/1	0/1	0/1
22	*Proteus mirabilis* MTCC 3310	0/1	1/1	0/1	0/1	0/1	0/1	0/1	0/1	0/1
23	*Citrobacter freundii* ****ATCC 8090	0/1	1/1	0/1	0/1	0/1	0/1	0/1	0/1	0/1
24	*Enterobacter aerogenes* ATCC 13048	0/1	1/1	0/1	0/1	0/1	0/1	0/1	0/1	0/1
25	*Salmonella arizonae* ATCC 13314	0/1	1/1	0/1	0/1	0/1	0/1	0/1	0/1	0/1
26	*Klebsiella pneumoniae* ATCC 13883	0/1	1/1	0/1	0/1	0/1	0/1	0/1	0/1	0/1
27	*Shigella flexneri* ATCC 9199	0/1	1/1	0/1	0/1	0/1	0/1	0/1	0/1	0/1
28	*Shigella sonnei* ATCC 25931	0/1	1/1	0/1	0/1	0/1	0/1	0/1	0/1	0/1
29	*Shigella boydii* ATCC 9207	0/1	1/1	0/1	0/1	0/1	0/1	0/1	0/1	0/1
30	*Yersinia enterocolitica* ATCC 23715	0/1	1/1	0/1	0/1	0/1	0/1	0/1	0/1	0/1

* NCTC - National Collection of Type Cultures, Salisbury, UK.

**MTCC - Microbial Type Culture Collection, Chandigarh, India.

***NCIM - National Collection of Industrial Microorganisms, Pune, India.

****ATCC - American Type Culture Collection, Manassas, USA.

### Cell Line

Human alveolar adenocarcinoma epithelial cells - A549, were procured from National Cell Centre for Science, (Pune, India) and grown in Dulbecco's modified Eagle medium (Sigma, India) supplemented with 10% fetal bovine serum (FBS) (Sigma, India) and 1× working concentration of Penicillin-Streptomycin solution (Sigma, India) at 37°C in a humidified atmosphere of 5% CO_2_, as recommended by the supplier. The cells were seeded in tissue culture flasks (Greiner bio-one, US) and allowed to grow to 80–90% confluency. Cells were dislodged with 0.025% (w/v) trypsin/0.01% (w/v) EDTA (Sigma, India), counted using a heamocytometer and seeded into 24 well plates (5×I0^5^ cells/well) for invasion and apoptosis inhibition assays; and 6 well plates (1×10^6^ cells/well) containing sterile 12 mm diameter glass coverslips coated with 0.1% gelatin for immunofluorescence assay. Cells were incubated to reach 80–90% confluency at 37°C in a humidified atmosphere of 5% CO_2_.

### Outer Membrane Proteins Preparation

OMPs were extracted from *Burkholderia pseudomallei* NCTC 10274 as described by Crosa and Hodges [28]. Briefly, the bacterium was grown overnight aerobically in 200 ml of LB broth and cells were harvested by centrifugation at 5000× *g* for 30 min at 4°C. Harvested cells were re-suspended in 3 ml of 10 mM Tris amino methane buffer containing 0.3% (w/v) NaCl (pH-8.0). The suspension was treated with 1% (w/v) thiomersol and 1 mM phenylmethyl sulfonyl fluoride (PMSF) and sonicated with a Vibra cell sonicator (Sonics & Materials Inc., Newtown, CT) for 3 times at 10 µM amplitude for 15 minutes with 30 sec interval for every 5 min. Lyzed suspension was centrifuged at 10000× *g* for 2 min and the supernatant collected was further centrifuged for 1 h at 17000× *g* at 4°C. The pellets of cell envelop suspension thus obtained were incubated overnight at 4°C with 3% (w/v) sodium lauroyl sarcosinate (sarkosyl) in 10 mM Tris buffer. OMPs were obtained by centrifugation at 17000× *g* for 1 h and washed twice with distilled water. Resulting OMPs pellet was re-suspended in sterile 10 mM Tris amino methane buffer containing 0.3% NaCl (pH-8.0) and concentration of protein preparation was quantified by Lowry method [29]. The outer membrane proteins profile was analyzed by SDS-PAGE according to standard procedure by Laemmli *et al* [30].

### Immunization

Two female BALB/c mice (one six weeks old weighing 19 g and one eight weeks old weighing 20 g) were subcutaneously immunized with purified crude OMPs of *Burkholderia pseudomallei*. Briefly, primary immunization was done with 100 µg of purified OMPs in emulsion with Freund's complete adjuvant (Sigma, USA). Two booster doses of 50 µg protein with Freund's incomplete adjuvant (Sigma, USA) were followed on days 14 and 28. The serum was collected from mice prior to each immunization and stored at −20°C. The antibody titer of immunized mice sera was measured by indirect plate ELISA using crude OMPs as antigen. Upon confirmation for desired titer, a final immunization with 100 µg of the antigen in 1× sterile phosphate-buffered saline (7 mM Na_2_HPO_4_, 3 mM NaH_2_PO_4_ and 130 mM NaCl at pH 7.4) i.e., PBS, intraperitoneally was done a week prior to sacrifice for generation of hybridomas.

### Mouse Monoclonal Antibodies Generation

Hybridoma fusions were performed to generate monoclonal antibodies (mAbs), according to Kohler and Milstein [31]. Briefly, upon achievement of the desired antibody titer, spleen cells from immunized BALB/c mice were fused with sp2/0-Ag.14-1 cells (ATCC, VA) using 50% w/v PEG MW 2000 (Sigma, India). Fused cells were grown in selective HAT-containing Dulbecco's modified Eagle's media (DMEM, Sigma, India) and 10% v/v fetal bovine serum (Sigma, India). The generated hybrid clones were screened for their reactivity against the antigen by indirect plate ELISA. Promising hybridomas were further cloned by limiting dilution method in 96 well tissue culture plates (Greiner bio-one, US) and resulting specific hybridomas were subjected to expansion. Followed by final expansion, specific hybridomas were temporarily frozen in duplicate vials.

### Characterization of mAbs

- **Analysis for specificity.** An indirect plate ELISA was performed to ascertain reactivity and specificity of mAbs with the crude OMPs preparation and/or whole cell lysates of strains listed in Table 1. ELISA plates were coated with 100 µl/well of crude OMPs or whole cell lysate preparation at a concentration of 20 µg in each milliliter of 50 mM carbonate/bicarbonate buffer (pH 9.6) for 1 h at 37°C and blocked with 1% (w/v) bovine serum albumin (BSA) (Sigma, India) in 1× PBS. After each step, plates were washed three times with 1× PBST (0.05% of Tween 20). Specificity of reactive hybridomas was assessed by incubating the plates with 100 µl of hybridoma culture supernatant containing mAbs for 1 h at 37°C. Horse radish peroxidase (HRP) conjugated goat-anti-mouse-IgG was used as secondary antibody and developed using O-phenylene dihydrochloride, as substrate. Mice anti-OMPs serum diluted at 1/1000 was used as positive control. The average absorbance of replicate wells was used after subtraction of background (BSA-blocked wells). ELISA end-point titers were defined as the serum dilution that gave 20% of maximum A_450_ nm value.
- **Determination of biomolecular nature of epitope bound by mAbs.** Two promising mAbs: BURK24 and BURK37 (discussed in results section) were subjected to Western blots as described [32] to determine the antigen-antibody binding pattern and biomolecular nature of epitope. Either crude OMPs preparation or whole-cell bacterial lysate of *B. pseudomallei* NCTC 10274 was used as antigen. Lysate was prepared by boiling bacteria, grown on blood agar plates, in Laemmli sample buffer. Proteins were separated on sodium dodecyl sulphate-polyacrylamide gels by electrophoresis, electroblotted onto nitrocellulose paper, probed with mAbs: BURK24 and BURK37, followed by horse radish peroxidase-conjugated goat anti-mouse IgG (Sigma, India). Blots were developed with 0.3% diaminobenzidine tetrahydrochloride (Sigma, USA) in 70 mM sodium acetate, pH 6.2 or 3,3′,5,5′ tetramethylbenzidine with 0.03% H_2_O_2_ (v/v) (Sigma, USA) and signals were captured by luminescent image analyzer (Fujifilm, India). In order to determine the biomolecular nature of epitope, an aliquot of OMPs preparation was subjected to protein digestion using 0.02 unit of proteinase K for 30 min at 55°C. Resulting antigen was immunoblotted as described above.
- **Isotyping.** Isotyping of mAbs BURK24 and BURK37 was performed using commercially available ELISA based isotyping kit (Sigma, USA) as described by the manufacturer. Briefly, 96-well immunosorbent plates (Nalge Nunc International, NY) were coated with specific goat anti-mouse immunoglobulin Fc subclass antibodies and blocked with 1% bovine serum albumin (BSA). Hybridoma culture supernatants were added and incubated in the plates for 1 h at room temperature. Horseradish peroxidase (HRP)-conjugated detection antibodies specifically against each mouse immunoglobulin Fc subclass were then added and incubated for 1 h. The isotype of each mAb was revealed using O-phenylene dihydrochloride with 30% H_2_O_2_ as HRP substrate.

### Purification of mAbs

In order to obtain large concentrations of mAbs, for further studies, ascitic fluids of monoclonal antibodies BURK24 and BURK37 were generated produced in 2,6,10,14-tetramethyl pentadecane i.e., Pristane (Sigma, India) primed retired breeder BALB/c mice, by injecting BURK24 and BURK37 hybridomas as described by R B Westerman *et al.* [33] and purified using mAbTrap kit and HiTrap IgM Purification HP (GE Healthcare, UK) respectively as per the manufacturer's instructions. The purified mAbs were lyophilized, quantified and reconstituted in sterile 1× PBS according to the required concentrations of each study.

### Indirect Immunofluorescence Binding Assay

The binding specificity of BURK24 and BURK37 mAbs onto the surface of invading *B. pseudomallei* NCTC 10274 into a human alveolar epithelial carcinoma cell line A549, was deduced by indirect immunofluorescence binding assay. Briefly, A549 mammalian cells were grown on to the 12 mm 0.1% gelatin coated glass coverslips in 6 wells tissue culture plates (Nalge Nunc International, NY). Cells were grown overnight in DMEM tissue culture medium supplemented with 10% FBS till 80–90% confluency before performing the assay. The coverslips coated with A549 mammalian cells were washed thrice with 1× sterile PBS and infected with overnight grown culture (OD at 600 nm of 0.6) of *B. pseudomallei* NCTC 10274 at a multiplicity of infection (MOI) of 50∶1. After 30 min of incubation, the coverslips were washed with 1× PBS and air dried. Infected cells on to the coverslips were fixed with 3.7% formaldehyde in 1× PBS for 15 min. One percent BSA (Sigma, India) was employed for blocking the cells for duration of 30 min to minimize non-specific binding of antibodies before proceeding further. The formaldehyde fixed cells were incubated with 50 µg of mAb BURK24 and BURK37 separately at 37°C for 1 h. Later, the coverslips were washed twice with 1× sterile PBS, air dried and further incubated for 1 h with 1 ml of 1∶500 dilution of anti-mouse immunoglobulin in goat, conjugated with fluorescein isothiocyanate (FITC) (Sigma, USA) at RT as recommended by the manufacturers. Again the coverslips were washed twice with 1× sterile PBS; air dried and coated with Flouroshield (Sigma, USA) for microscopic examination of BURK24 and BURK37 associated bacteria onto the surface of infected A549 human alveolar epithelial carcinoma cell line under fluorescence microscope (Nikon Eclipse Ni, Japan). Non-invasive strain of *E. coli*, control serum from naive BALB/c mouse and conjugate alone were used as negative controls in the experiment.

### *In vitro* Antimicrobial Studies

- **Antibody susceptibility test.** To examine the antimicrobial property of BURK24 and BURK37, microtiter-based minimum inhibitory concentration (MIC) and minimum bactericidal concentration (MBC) tests were done as described in guidelines for antimicrobial susceptibility testing [34], [35] by CLSI. Briefly, serial two-fold dilutions of purified monoclonal antibodies were performed using MH broth from a stock solution of 1 mg/ml concentration in a flat bottomed 96 wells microtiter plate (Nalge Nunc International, NY), resulting in doubling dilutions throughout the microdilution tray. Suspensions containing 5×10^5^ CFU/ml of exponentially growing *B. pseudomallei* NCTC 10274 and *B. pseudomallei* strain 621 were added to separate wells containing mAb dilutions, such that the total reaction mixture contained 100 µl of mAb and 100 µl of bacteria and incubated aerobically at 37°C with vigorous shaking at 250 rpm. Prior to incubation, growth control wells were sampled and plated to confirm the initial inocula. The lowest concentration of mAb in which there was no visible growth after overnight incubation was recorded as MIC. MBCs were determined by sub culturing 10 µl of reaction mixture at appropriate dilution, from wells where there was no visible growth, on MH agar plates and incubated at 37°C for colony count. MBC was defined as the lowest dilution showing ≥99.9% kill or a 3 log decrease in the CFU/ml after 24 h of incubation. The ratio of MBC to MIC was determined for both mAbs.
- **Bacteriostasis: Kinetic study.** To determine the kinetics of bacteriostasis of both the mAbs: BURK24 and BURK37, microplate based bacteriostatic assay was designed and performed using ELISA plate reader (InfiniteM200, Tecan, Austria). An aliquot of 100 µl of exponentially grown *B. pseudomallei* NCTC 10274, adjusted to a bacterial load of 5×10^5^ CFU/ml was added to the equal volume of mAb dilutions-prepared as in determination of MIC and MBC, from 1 mg/ml to 15 µg/ml in microtiter plate (Nalge Nunc International, NY) and the mixture was mixed thoroughly by shaking the plate for 10 min at 250 rpm and the absorbance was measured at 595 nm and it was considered as the absorbance at 0 h. The plate was further incubated in the plate reader at 37°C with constant agitation at 250 rpm and absorbance (OD_595_) was recorded at equal intervals of 1 h for eight consecutive hours. Wells containing 100 µl MH broth mixed with 100 µl of 5×10^5^ CFU/ml of *B. pseudomallei* NCTC 10274 and MH broth without mAb were used as controls.
- **Time-kill curve analysis.** Time-kill studies of bactericidal concentrations of BURK24 and BURK37 were performed in microdilution method according CLSI guidelines [35] to study the lethal activity of BURK24 and BURK37 and also to determine whether the bacterial killing is concentration and/or time dependent. The experiments were performed with exponential culture of *B. pseudomallei* NCTC 10274, diluted to a load of 5×10^5^ CFU/ml and a bactericidal concentration of mAb. Lethal activity may be expressed as the rate of killing by a fixed concentration of antimicrobial under controlled conditions. This rate is determined by measuring the number of viable bacteria at various time intervals. The resulting graphic depiction is known as the time-kill curve. Briefly, 50 µl of MHB containing approximately 5×10^5^ CFU/ml of *B. pseudomallei* standard strain was taken in each well of a microtiter plate and mixed with of 50 µl of 1× sterile PBS containing of bactericidal concentration of mAb, of BURK24 and BURK37 respectively. A negative growth control containing 100 µl of MHB with 5×10^5^ CFU/ml of *B. pseudomallei* NCTC 10274 and no mAb was included. At intervals of 0, 4, 8, 10 to 12, and 24 hours incubation, samples were drawn, serially diluted and appropriate dilution was plated on MHA plate to get countable colonies. The lower limit of accuracy of bacterial counting was 300 CFU/ml. The colony count data in terms of log10 CFU/ml from triplicate time–kill studies were averaged and plotted as a function of time. For time–kill experiments, cutoff point of bactericidal activity was defined as a decrease in CFU by 3 log10 CFU/ml after treatment for 24 h.

### Effect of mAbs on Biofilm Formation

The amount of biofilm inhibition induced by the mAbs of the study was determined by a semi-quantitative microtiter plate based assay as described elsewhere [36], [37] with few modifications. An overnight culture of *B. pseudomallei* standard strain grown at 37°C in TSB with 50 mM glucose was adjusted to a bacteria load of 5×10^5^ CFU/ml and 100 µl of the culture was mixed with equal volume of earlier deduced bacteriostatic concentrations of BURK24 and BURK37. This mixture was transferred to a flat-bottomed microtiter plate and incubated aerobically at 37°C for 24 h without shaking. After incubation period, the culture was removed and microtiter plate was washed three times with 1× sterile PBS to remove non adherent bacterial cells and dried in inverted position. Adherent dry biofilm was fixed with 250 µl of methanol for 15 min. The wells were then emptied and air dried. For staining, 250 µl of 1% (w/v) crystal violet was added to each well and incubated for 5 min. The wells were then washed under running tap water and air dried. The adherent bacterial cells in the wells were re-solubilized by adding 250 µl of 33% (v/v) glacial acetic acid per well. The optical density (OD) of each well was measured at 570 nm using an automated ELISA plate reader (InfiniteM200, Tecan, Austria). A negative control containing *B. pseudomallei* standard strain in TSB with 50 mM glucose in the absence of mAbs was included. The assay was performed twice in triplicates. With the obtained OD values, percent inhibition of biofilm formation was also determined using the following formula.
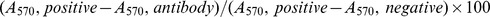



### Invasion Inhibition Assay

The ability of BURK24 and BURK37 to inhibit the invasion of *B. pseudomallei* into a human alveolar epithelial carcinoma cell line A549 was examined in this experiment. A549 cells were grown in 24-well tissue culture plates containing DMEM tissue culture medium supplemented with 10% FBS. The assay was performed in triplicate using *B. pseudomallei* NCTC 10274 (OD at 600 nm of 0.6) mixed with BURK24 and BURK37 separately at earlier determined bacteriostatic concentration. The bacterial cells were harvested from the mixture after 30 min, re-suspended in 200 µl of DMEM tissue culture medium supplemented with 2% FBS and transferred to the 24-well tissue culture plates at an MOI of 50∶1. The infected confluent monolayers of eukaryotic cells were incubated at 37°C in a humidified atmosphere of 5% CO_2_ for 1 h to allow bacterial entry. After invasion, all the wells were washed with 1× sterile PBS for three times and incubated with DMEM supplemented with 2% FBS containing 150 µg/ml tetracycline for 2 h at 37°C in a humidified atmosphere of 5% CO_2_ to kill the extracellular bacteria. After incubation, the cell monolayers were washed thrice with 1× sterile PBS and lyzed with 0.25% Triton X-100. Enumeration of the intracellular bacteria was done by plating the serial dilution of the lysate on TSB containing 4 mg/L of gentamicin and percentage of invasion in each case was determined using the formula given below. A positive control of non invasive *E. coli* strain was used in the experiment and in the negative control; monolayers of eukaryotic cells were infected with *B. pseudomallei* NCTC 10274 in the absence of mAbs. All quantitative invasion inhibition assays were performed in triplicate. The formula to determine percentage of invasion is as follows:
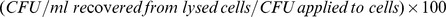



### Assessment of Cell Apoptosis

BURK24 and BURK37 were evaluated for their role in protection of A549 cell lines from *B. pseudomallei* infections by assessing the pathogen-induced apoptosis. The assay was performed using *B. pseudomallei* NCTC 10274 priorly incubated with each mAb separately at 4°C for 1 h. A549 cells were seeded at 5×I0^5^ cells/well in 24 well plates and incubated for 18 to 20 h prior to infection. The cells infected with *B. pseudomallei* at an MOI of approximately 50∶1 were included in the experiment to study the potency of the bacterium to induce apoptosis and compare the reduction in apoptosis in presence of the mAbs. After 1 h of infection, the cells were washed and further incubated in the presence of 150 µg/ml of tetracycline for 2 h at 37°C. The cells were washed thrice with 1× sterile PBS after an incubation period of 24 h and visualized under phase contrast microscope (Nikon Eclipse Ni, Japan). The study was repeated simultaneously to evaluate the effect of BURK24 and BURK37 on the DNA damage as well. At time intervals of 0 h, 2 h, 4 h, 6 h, 8 h, 12 h, 16 h, 20 h and 24 h, A549 cells were washed, trypsinized, pelleted and lyzed in buffer containing 10 mM Tris-HCl, pH 8.0–100 mM NaCl–0.5% SDS–35 mM EDTA. The lysate was then treated with proteinase K (0.1 mg/ml) at 50°C for 2 h. Protein was removed by extraction with phenol-chloroform-isoamyl alcohol (25∶24∶1). Nucleic acids were then precipitated using ethanol and centrifuged at 9500× *g* for 30 min. The DNA pellets were air dried; re-suspended in 1× TE buffer (10 mM Tris-HCl, pH 8.0–1 mM EDTA) containing RNase (0.1 mg/ml) and subjected to electrophoresis in 1.8% Seakem LE agarose (Lonza) gel. The gel was then stained with ethidium bromide and visually examined under UV light at 305 nm. Only A549 cells and A549 cells infected with *B. pseudomallei* NCTC 10274 in the absence of mAbs, were included in the experiment as negative and positive control respectively.

### Statistical Analysis

Statistical analyses were performed using Multivariate Analysis of Variance (MANOVA) and Repeated measure Analysis of Variance (RANOVA) to analyze statistical difference exhibited by *B. pseudomallei* in terms of growth and biofilm formation in presence and absence of BURK24 and BURK37. Bonferroni post hoc test was performed where applicable. Data are expressed as the mean ± standard deviation. Differences were considered statistically significant at a p-value≤0.05. All statistical analyses were performed using Statistical Package for Social Science (SPSS) version 16.

## Results

### Immunization and Generation of mAbs

**Figure 1:**
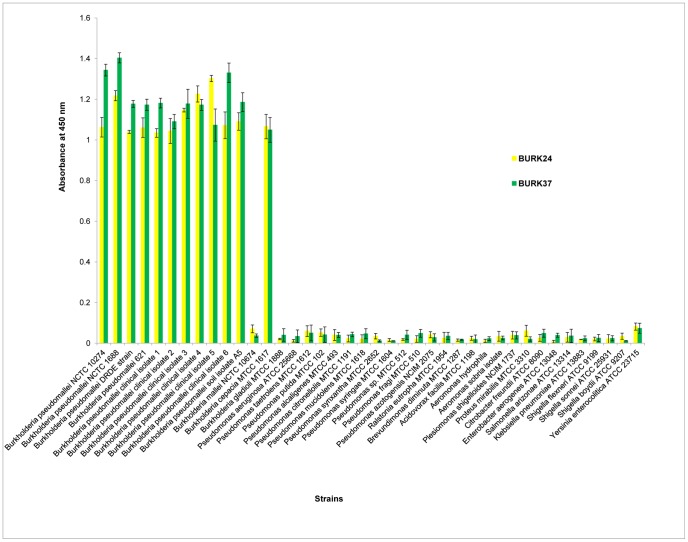
Specificity evaluation for BURK24 and BURK37. Culture supernatants of BURK24 and BURK37 hybridoma were evaluated for their specificity by indirect plate ELISA. Both the mAbs showed 100% specificity with a total of eleven strains of *B. pseudomallei* tested. Additional reactivity with *B. cepacia* MTCC 1617 of the mAbs demonstrated the sharing of their respective epitopes among both the species. Data in this figure is average of two independent experiments. All error bars represent the standard error.

Gradual increase in antibody titer was observed in BALB/c mice sera after each immunization, as revealed by indirect plate ELISA. Comparatively no reactivity was registered in the pre-immune serum against the outer membrane proteins. This indicated that the OMPs extract used was immunogenic resulting in antibody response in immunized mice. The desired antibody titer of 1∶64000 was achieved after second booster immunization. The polysera showed reactivity with crude OMPs extract, whole cell lysate and intact cells of *B. pseudomallei* NCTC 10274 (data not shown). This implied that the polysera harbored antibodies with ability to recognize native epitope. Since the reactivity of polysera was promising, a panel of monoclonal antibodies were generated using the sensitized spleenocytes from BALB/c mice by standard hybridoma technique. A total of one hundred and eight clonal hybridomas were initially screened by an indirect plate ELISA (data not shown) for their reactivity with the OMPs preparation. This resulted in narrowing down to a total of nine mAbs (Table 1). To elaborate, among nine mAbs, 6 demonstrated a reactivity of 9.09%, one mAb showed 72.72% reactivity and two mAbs designated as BURK24 and BURK37 demonstrated a reactivity of 100% with all the eleven strains of *B. pseudomallei* tested (Table 1, Figure 1). The two mAbs with 100% reactivity for *B. pseudomallei* strains tested additionally showed cross-reactivity with *B. cepacia* MTCC 1617 (Table 1, Figure 1). Hence the two mAbs were selected for assessment of their anti-*B. pseudomallei* properties.

### Characterization of mAbs

**Figure 2:**
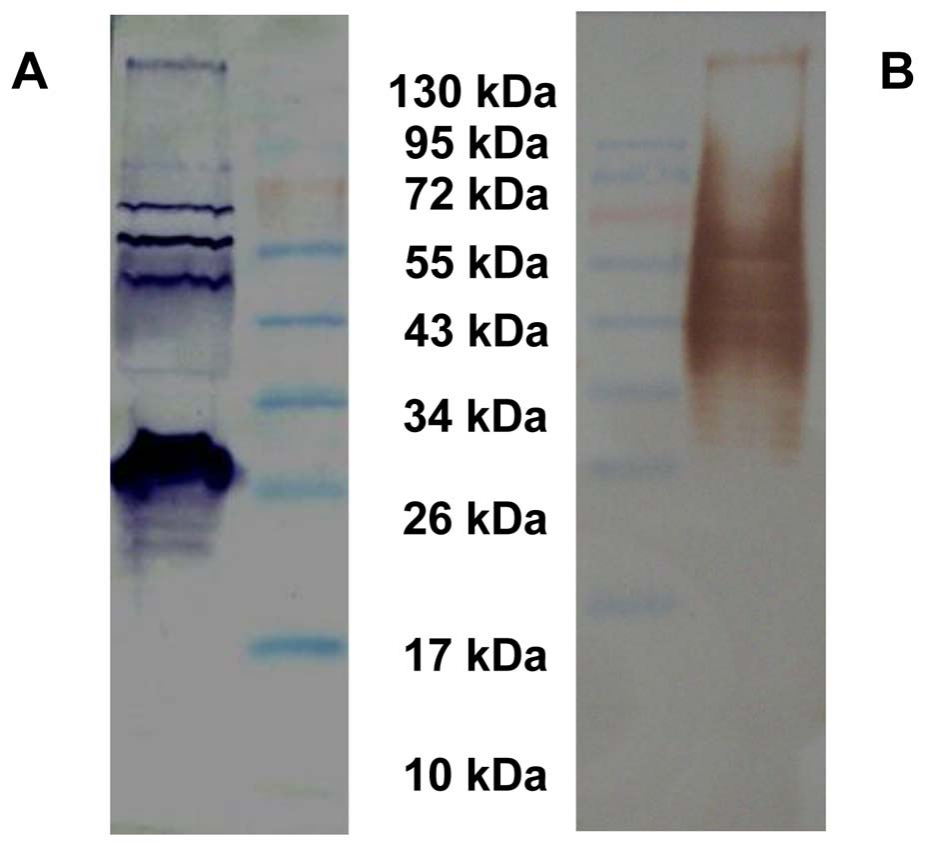
Determination of chemical nature of epitope of the mAbs-BURK24 and BURK37. (**A**) Western blot pattern of BURK24 with crude outer membrane proteins extract of *B. pseudomallei* NCTC 10274. The blotting pattern revealed protein bands at 70 kDa, 55 kDa, 50 kDa and 26 kDa. (**B**) Western blot of BURK37 showing a typical LPS ladder pattern with crude outer membrane proteins extract of *B. pseudomallei* NCTC 10274.

**Figure 3:**
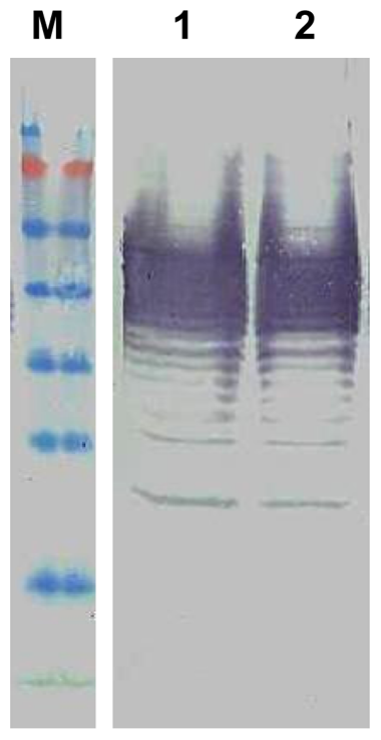
Determination of chemical nature of epitope for BURK37. Western blot analysis was performed for BURK37 using crude outer membrane proteins extract (lane 1) and the same antigen priorly subjected to complete protein digestion using 0.02 U of proteinase K for 30 min at 55°C (lane 2). The undistinguishable immunoblot pattern in both the lanes proves that the epitope for BURK37 is a non-proteinaceous compound. The experiment was repeated thrice for reproducibility and the typical LPS ladder pattern was obtained each time.

For better understanding about BURK24 and BURK37, Western blot analysis and isotyping were performed. Both the mAbs - BURK24 and BURK37, were able to recognize the antigen and the biomolecular nature of their epitope was determined in Western blot assay (Figure 2). BURK24 was found to react with protein antigens (approximately 70 kDa, 55 kDa, 50 kDa and majorly at 26 kDa), indicating the presence of the epitope in multiple copies. Whereas, BURK37 revealed a typical LPS-ladder pattern [38], [39], [40], [41] ranging from 34–95 kDa. Since the crude extract of outer membrane proteins is known to have lipopolysaccharide components of bacterial cell wall, it was speculated that the antibodies raised against OMPs might include the one which reacts with the lipopolysaccharide also. To evaluate that the epitope of BURK37 was non-protein, Western blot was carried out using the proteinase K digested crude OMPs of *B. pseudomallei* NCTC 10274. The resulting immunoblot pattern of both proteinase K treated and untreated revealed identical immunoblot pattern inferring that the epitope is non-proteinaceous compound, i.e., lipopolysaccharide which has contributed to give raise the mAb - BURK37 (Figure 3). Similar proteinase K treatment experiment study for BURK24 resulted in no immunoblot implying that its epitope is proteinaceous (data not shown). BURK24 and BURK37 were deduced to be IgG1 and IgM respectively upon isotyping. Higher concentrations of mAbs were obtained by producing ascitic fluids in BALB/c mice, purified, concentrated by lyophilization and quantified by Lowry method. The concentration of mAbs BURK24 and BURK37 was 2.5 and 2.3 mg/ml respectively.

### Interaction of BURK24 and BURK37 with *B. pseudomallei*

**Figure 4:**
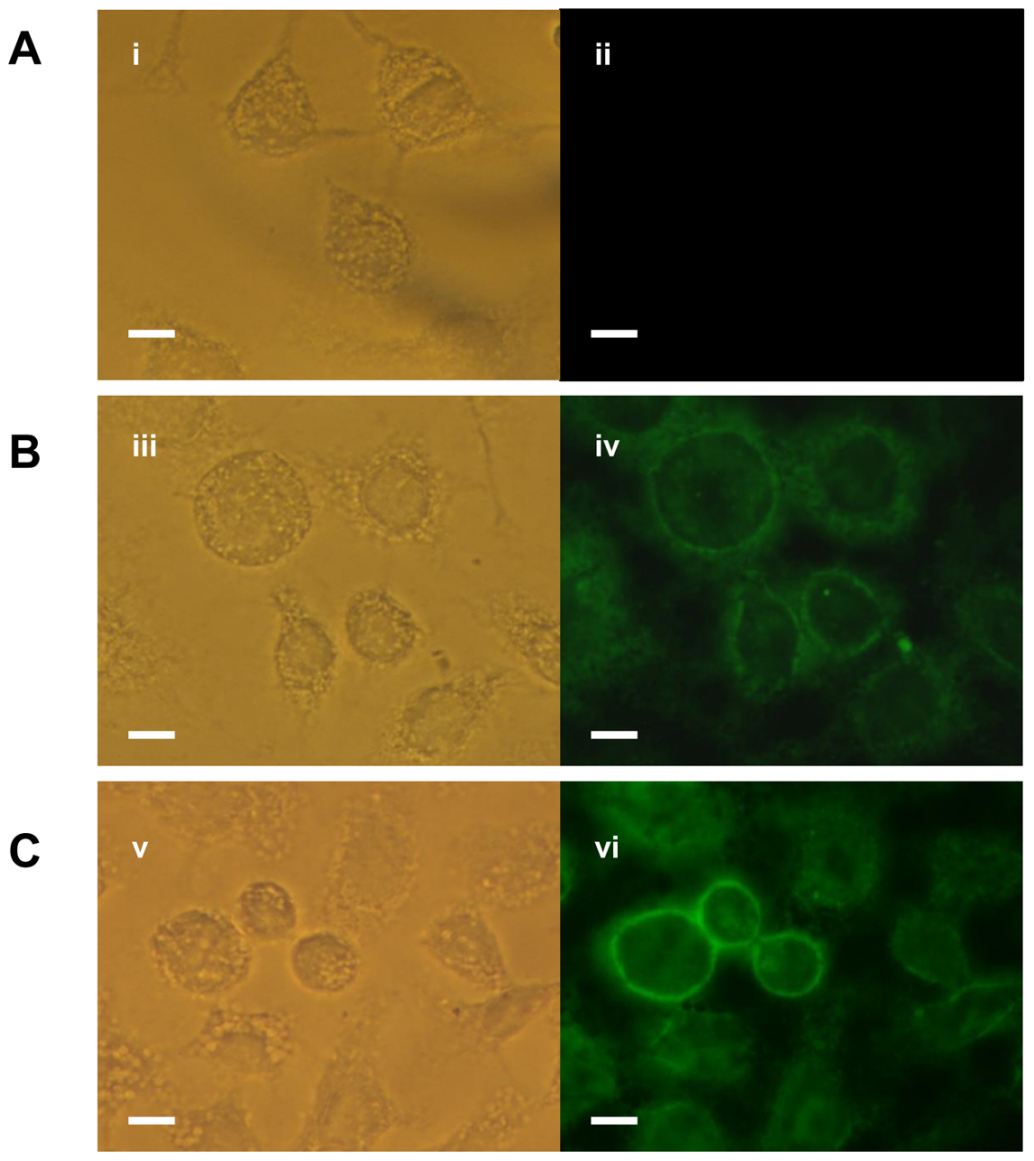
Indirect immunofluorescence binding assay of BURK24 and BURK37. Human lung adenocarcinoma A549 cells were seeded overnight onto 12×. (**A**) A549 cell infected with non-invasive *E. coli* cells followed by treatment with control serum from naive BALB/c mouse. (**i**) Phase contrast of A549 cells (**ii**) Absence of immunofluorescence indicating the failure of naive mouse serum to bind onto the non-invasive strain of *E. coli*. (**B**) A549 cells infected with *Burkholderia pseudomallei* NCTC 10274 followed by immunostaining using BURK24 as primary antibody and FITC conjugated anti-mouse immunoglobulin as secondary antibody. (**iii**) Phase contrast microscopic observation of A549 cells after immunostaining (**iv**) Fluorescence of the *B. pseudomallei* NCTC 10274 infected cell lines indicating the specific binding of BURK24 on live and intact bacteria. (**C**) A549 cell infected with *B. pseudomallei* NCTC 10274 followed by indirect immunofluorescence using BURK37. (**v**) A549 cells under phase contrast microscopic observation (**vi**) Fluorescence of the cell lines indicating the positive specific binding of BURK37 onto the bacterial cell surface as in case of BURK24. The results are representative of two independent experiments. Bar = 10 µm.

Before proceeding with the studies on determining the functional properties of BURK24 and BURK37 towards the anti-*B. pseudomallei* pathogenesis, the mAbs were confirmed for the interaction with intact *B. pseudomallei* cells by a novel indirect immunofluorescence based assay. Human alveolar epithelial carcinoma cell lines - A549 were employed in the assay for the direct visualization of the result. The processed coverslips when observed under microscope at 525 nm wavelength, a strong green visual signal at the periphery of A549 cells was recorded highlighting the *B. pseudomallei* infected host cells (Figure 4). BURK24 and BURK37 were found to specifically bind onto the invading *B. pseudomallei* NCTC 10274 on the A549 mammalian cell lines. In the context of indirect ELISA, BURK24 and BURK37 was found to be significantly consistent in terms of their reactivity and specificity. In this study, to prevent the loss of intactness of bacterial cells we have used mAbs in not more than bacteriostatic concentration and also permeabilizing reagents like Tween 20 were not used to retain the intactness of host cells. Resulting immunofluorescence when observed under fluorescent microscope in the presence of specific mAbs confirmed the surface localization of antigens on *B. pseudomallei* NCTC 10274. No fluorescence was observed when non-invasive strain of *E. coli*, control serum and conjugate alone were used as negative controls. This implied that the immunofluorescence was solely due to the specificity of the mAbs for *B. pseudomallei*.

### Antimicrobial Activity

Although the mAbs certified their ability to bind on the intact bacterial cells, we questioned whether this property rendered any antibacterial activity to them. To evaluate this, we assessed methodically the antimicrobial activity of purified mAbs on *B. pseudomallei*. Since, minimum inhibitory concentration (MIC) and minimum bactericidal concentration (MBC) determine the optimum bacteriostatic and bactericidal concentration of any antimicrobial agent we proceeded to ascertain the same for BURK24 and BURK37. An experiment was conducted according to the CLSI guidelines [34], [35] in microdilution method to deduce the concentrations of BURK24 and BURK37 for the assessment of their functional attributes as protective molecules. The MIC was read as the concentration of mAb at which there was no visible growth. The MBC was read as the concentration of mAb which resulted complete inhibition of bacterial growth upon plating. The results are shown in Table 2. A uniform susceptibility to the mAbs was demonstrated by both the strains of *B. pseudomallei* tested. Briefly, MIC of both the strains tested, *B. pseudomallei* NCTC 10274 and *B. pseudomallei* strain 621, was determined as 30 µg/ml and 62.5 µg/ml for BURK24 and BURK37, respectively. Whereas, MBC was 125 µg/ml and 500 µg/ml for BURK24 and BURK37, respectively. . The respective MBC∶MIC ratio of mAbs: BURK24 and BURK37 was 4.1 and 8, indicating a bacteriostatic mode of action on *B. pseudomallei*. The experiment was repeated (n = 6) and the results were found to be statistically significant (p<0.05) and reproducible.

**Table 2:** Antibody susceptibility test for *B. pseudomallei* NCTC 10274 and *B. pseudomallei* strain 621 using BURK24 and BURK37.

mAb	MBC (µg/ml)		MIC (µg/ml)		MBC∶MIC		Inference
	*BPN	**BPC	BPN	BPC	BPN	BPC	
BURK24	125	125	30	30	4.1	4.1	Bacteriostatic
BURK37	500	500	62.5	62.5	8	8	Bacteriostatic

*B. pseudomallei NCTC 10274.

** *B. pseudomallei* strain 621.

**Figure 5:**
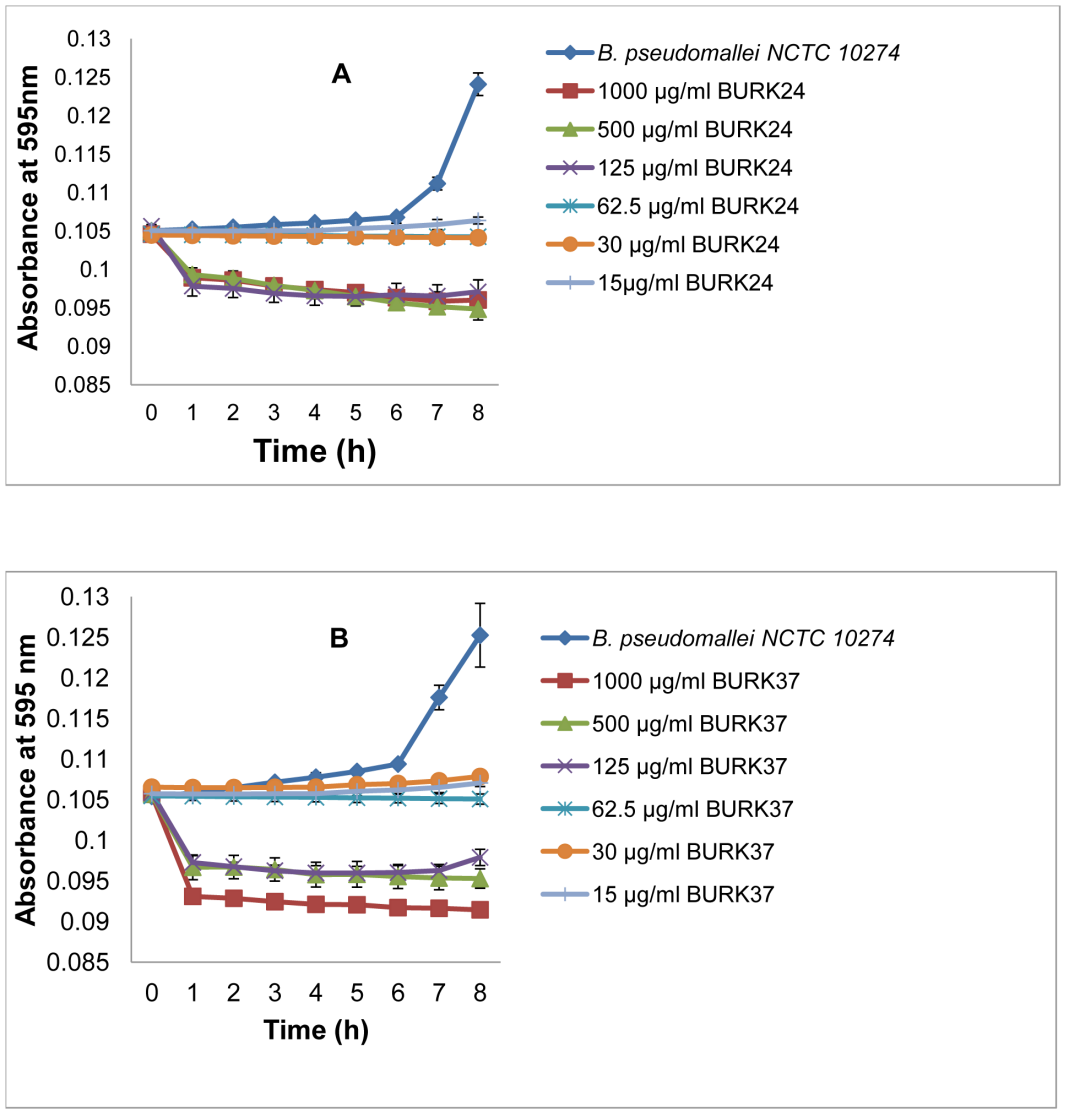
Kinetic study on bacteriostatic activity of BURK24 and BURK37 against *Burkholderia pseudomallei* NCTC 10274. BURK24 and BURK37 were examined spectrophotometrically at two-fold dilutions from 1 mg/ml to 15 µg/ml for their antibacterial activity for a duration of 8 h according to CLSI guidelines. (**A**) Kinetics of antibacterial activity exhibited by BURK24 on 5×10^5^ CFU/ml of *Burkholderia pseudomallei* NCTC 10274. (**B**) Antibacterial activity exhibited by BURK37 on the pathogen. In this study, both the mAbs exhibited antibacterial activity from 1 h onwards. Also, the respective static and cidal concentrations of BURK24 and BURK37 derived from this study were found to be concurrent to the results obtained in antibody susceptibility test. The error bar denotes the standard error. The reproducible bacteriostatic activity of BURK24 and BURK37 at concentrations of 30 µg/ml and 62.5 µg/ml respectively showed a statistically significant p-value of <0.05.

Our findings suggested that BURK24 and BURK37 possessed specific antibacterial activity. To further characterize this property of both the mAbs, we examined the kinetics of this activity. The study revealed that the range of mAb concentrations used in this experiment included bactericidal, bacteriostatic as well as non-bacteriostatic concentrations (Table 3). Upon comparison, the bacteriostatic and bactericidal concentrations derived in the kinetics study showed 100% concurrency with that of the antibody susceptibility testing. The kinetics study revealed that both the mAbs exhibited bacteriostatic activity within 1 h of the experiment (Figure 5). This advises that BURK24 and BURK37 might be promising in conferring immediate immunity in infected persons upon administration. The results also suggested that the bacteriostatic activity of BURK24 and BURK37 was concentration dependent as substantiated by statistical analysis. Bacteriostatic activity of BURK24 was statistically significant (p<0.05) at concentrations of 30 µg/ml and 62.5 µg/ml and insignificant (p = 1.000) at the concentration of 15 µg/ml. In case of BURK37, the activity was significant (p<0.05) at concentration of 60 µg/ml and insignificant (p = 1.000) at lower concentrations.

**Table 3:** Antibacterial concentrations of BURK24 and BURK37.

*B.pseudomallei* NCTC 10274					
BURK24 concentration (µg/ml)			BURK37 concentration (µg/ml)		
Uneffective	Bacteriostatic	Bactericidal	Uneffective	Bacteriostatic	Bactericidal
15	30	125	15	62.5	500
	62.5	500	30	125	1000
		1000			

**Figure 6:**
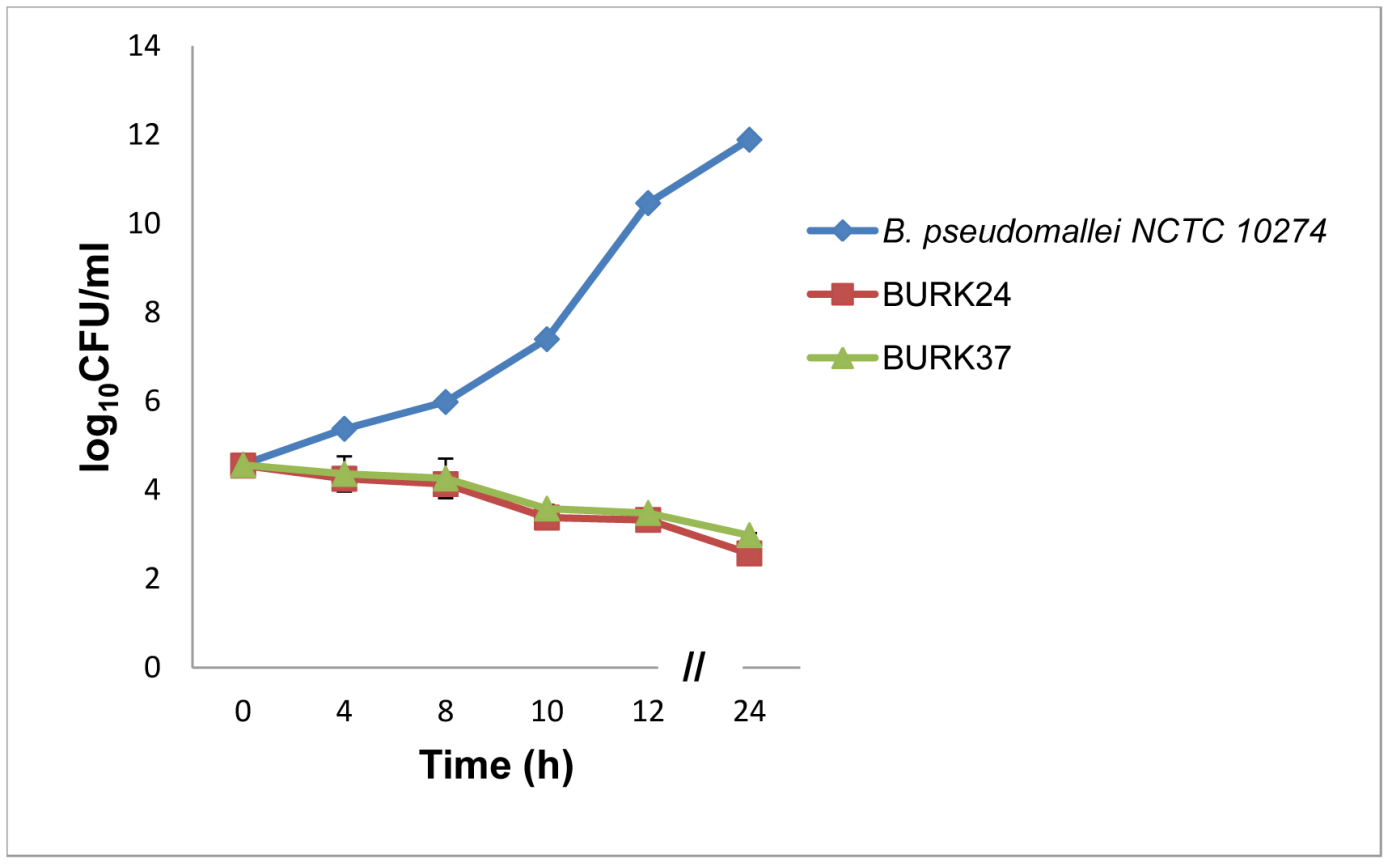
Determination of time-kill curve. Time based cidal activity of BURK24 and BURK37 on 5×10^5^ CFU/ml of *B. pseudomallei* NCTC 10274 was derived using their respective bactericidal concentrations as determined by antibody susceptibility test and bacteriostatic kinetics according to CLSI guidelines. Both the mAbs exhibited killing activity i.e., 3 log_10_ CFU/ml reduction in bacterial count after 4 h of the study and the killing effect persisted throughout the 24 h experimental duration. The log10 CFU/ml data presented is representative average of time-kill study performed in triplicates. The error bar denotes the standard error. The killing effect of BURK24 and BURK37 was found to be reproducible and statistically significant (p<0.05).

As described earlier in antibody susceptibility test and bacteriostatic kinetic study, BURK24 and BURK37 exhibited bactericidal activity at the concentration of 125 µg/ml and 500 µg/ml respectively. Here, we also studied the kinetics of bacterial killing exhibited by BURK24 and BURK37 *in vitro* to determine their respective time kill curve. The experimentally measured growth curves for *B. pseudomallei* NCTC 10274 in presence of 125 µg/ml of BURK24 and in presence of 500 µg/ml BURK37 in comparison with *B. pseudomallei* NCTC 10274 alone demonstrated significant reduction in growth. The gradual decrease in bacterial load was observed with the increase in time indicating that the killing effect of both the mAbs was time-dependent. And the 3 log10 CFU/ml reduction, which represents the 99.9% killing of bacteria was recorded after incubation for more than 4 h. The curve pattern revealed that both the mAbs demonstrated the bactericidal activity and the effect was persistent throughout the test duration of 24 h (Figure 6). The killing effect by BURK24 and BURK37 was confirmed to be reproducible and statistically significant (p<0.05) as well. Inspite of their difference in isotype, both the mAbs exhibited similar antimicrobial activity.

### Biofilm Inhibition Study

**Figure 7:**
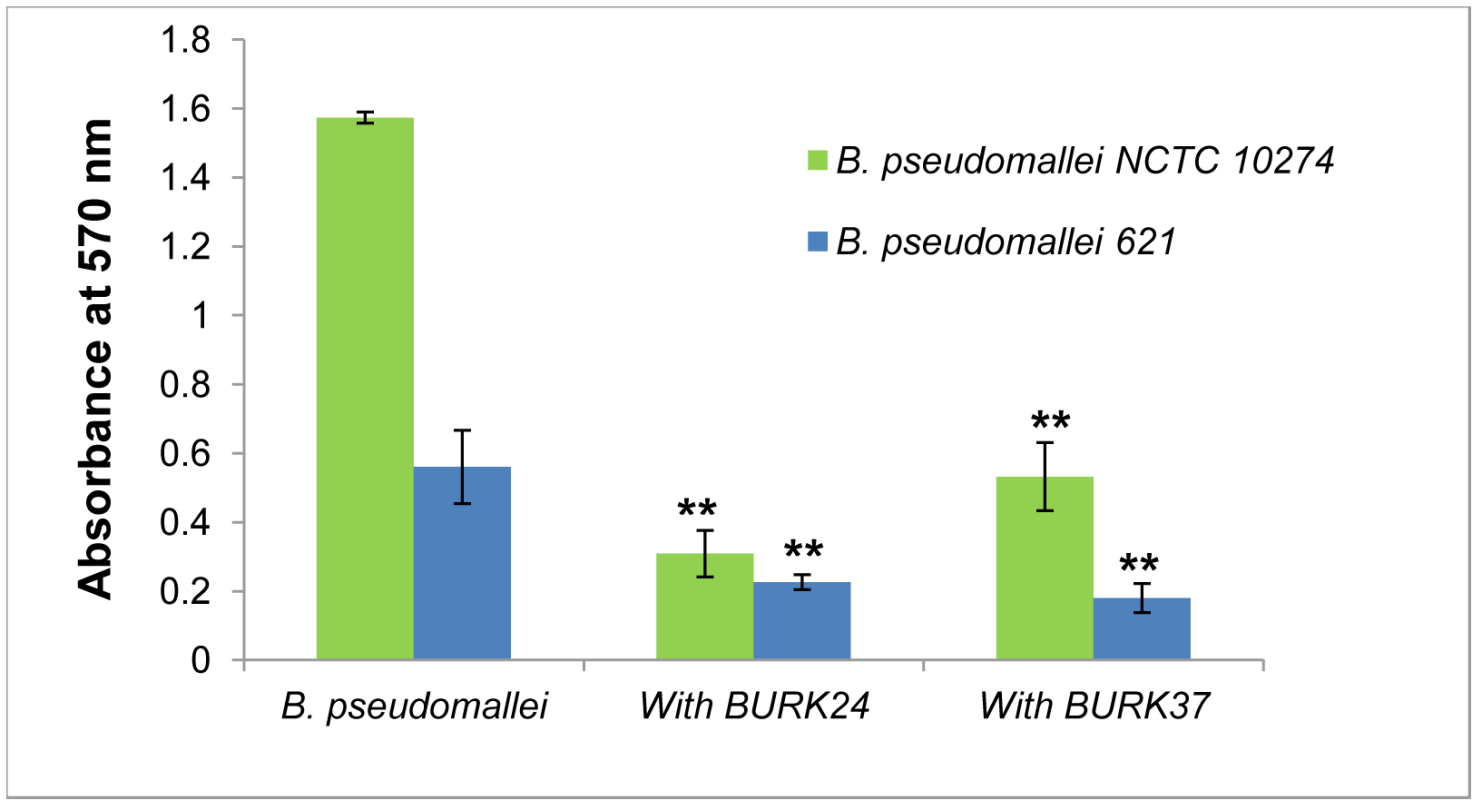
Quantification of biofilm formation in absence and presence BURK24 and BURK37. 5×10^5^ CFU/ml of *B. pseudomallei* NCTC 10274 and *B. pseudomallei* strain 621 were separately suspended in TSB with 50 mM glucose containing respective bacteriostatic concentration of BURK24 and BURK37 and incubated for 24 h at 37°C. Formation of biofilm was quantified with crystal violet stain. Both mAbs showed varied but considerable percentage of inhibition on biofilm formation capability of the strains studied. The graphical data presented here is average (n = 6) of A570 nm of the experiment. The error bar represents standard error.

**Figure 8:**
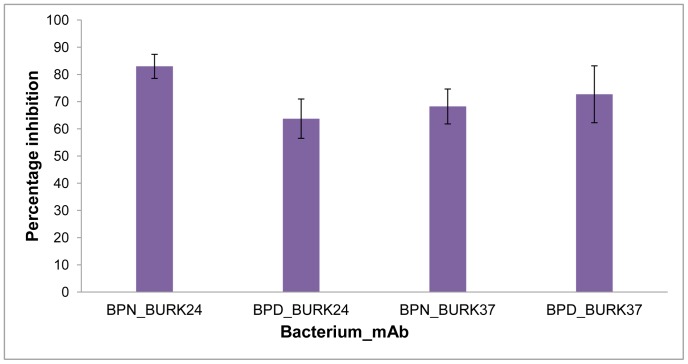
Average percentage inhibition of biofilm formation. Semi-quantitative biofilm inhibition assay was performed using *B. pseudomallei* NCTC 10274 (BPN) and *B. pseudomallei* strain 621 (BPD) in microtiter plate. Bacteria were grown in TSB medium supplemented with 50 mM of glucose as a biofilm formation inducer for 24 h at 37°C in presence of bacteriostatic concentration of BURK24 and BURK37. The amount biofilm formed was quantified and percentage of inhibition was further estimated. 5×10^5^ CFU/ml of *B. pseudomallei* NCTC 10274 in TSB with 50 mM glucose was used as control. The data presented is representative average of three repetitive experiments performed in duplicates. The error bar denotes the standard error.

As an analogous study to unravel the anti-*B. pseudomallei* based functional property of BURK24 and BURK37, we evaluated both the mAbs for their potential in inhibiting the formation of biofilm by *B. pseudomallei*. Since the biofilm forming potency varies among strains, both *B. pseudomallei* NCTC 10274 and the *B. pseudomallei* strain 621 were employed in the study in order to ascertain the inhibitory effect of BURK24 and BURK37 on variable biofilm formers. To begin with this, a microplate based assay was performed as described previously [36]. The rate of biofilm formation was estimated based on the OD_570_ (Figure 7) of the test samples and catagorized as highly positive (OD_570_≥1), low-grade positive (0.1≤OD_570_<1), or negative (OD_570_<0.1). *B. pseudomallei* NCTC 10274 was determined as a highly positive biofilm former with a mean OD_570_ of 1.57 whereas the *B. pseudomallei* strain 621 demonstrated a low-grade biofilm formation with a mean OD_570_ of 0.56. The average percentage of inhibition of biofilm formation (Table 4, Figure 8) exhibited by BURK24 on *B. pseudomallei* NCTC 10274 and *B. pseudomallei* strain 621 was recorded to be 82.95% and 63.72% respectively. Whereas, that of BURK37 on *B. pseudomallei* NCTC 10274 and *B. pseudomallei* strain 621 was 68.21% and 76.02% respectively. Also, statistical analysis based on OD_570_ of both the strains in presence and absence of individual mAb revealed significant difference (p<0.05). The results were found to be significantly reproducible upon repetition (n = 6). Though BURK24 and BURK37 exhibited variable activity as biofilm inhibitors on the two strains tested, overall effect was found to be significant.

**Table 4:** Biofilm inhibition by BURK24 and BURK37 on *B. pseudomallei* strains.

mAb	Biofilm inhibition (%)*	
	*B. pseudomallei* NCTC 10274	*B. pseudomallei* strain 621
**BURK24**	82.95±4.4	63.72±7.2
**BURK37**	68.21±6.4	76.02±6.1

*The percentage of biofilm inhibition is average of two independent experiments performed in triplicates.

### Blockade of Invasion

**Figure 9:**
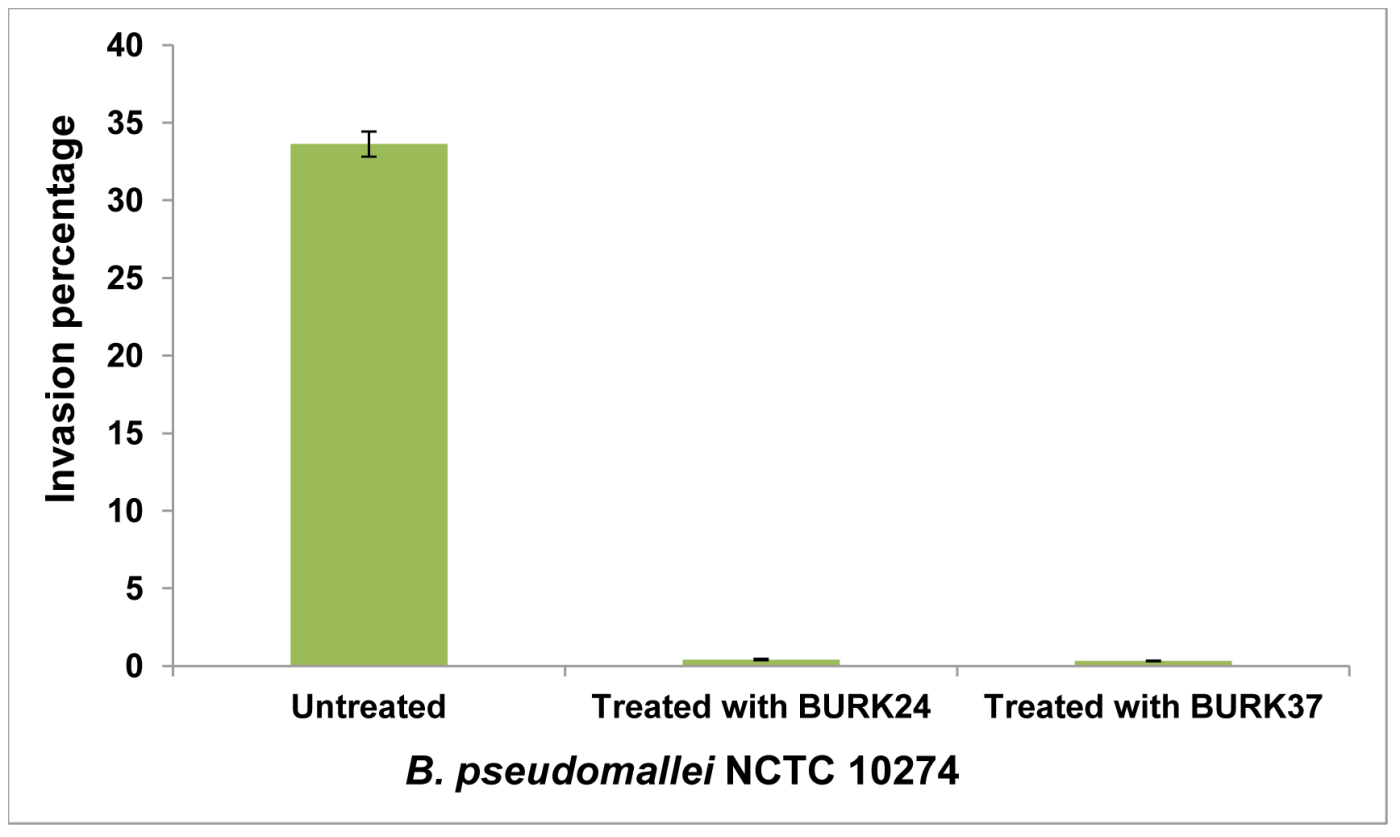
Percentage of invasion of *B. pseudomallei* NCTC 10274 in presence and absence of mAbs. Human alveolar epithelial cells-A549 were infected with *B. pseudomallei* NCTC 10274 at MOI of 50∶1 for a duration of 1 h. After infection period, non-invaded bacterial cells were killed using 150 µg/ml of tetracycline and A549 cells were permiabilized with 0.25% Triton X-100 to determine the percentage of bacteria invaded. The strain registered an average invasion rate of 33.46%. However, BURK24 and BURK37 inhibited the invasion of bacteria into A549 cells, resulting in a drastic decrease in percentage invasion to 0.41 and 0.32 respectively. The error bar denotes the standard error.

For the first time we have evaluated the inhibitory role of mAbs on invasion of *B. pseudomallei* into the epithelial cell lines. Both mAbs BURK24 and BURK37 at their bacteriostatic concentration showed virtually 100% invasion inhibition after 2 h of infection. To explain, the mean invasion capacity of *B. pseudomallei* NCTC 10274 was predetermined as to be 33.46%. Whereas, a mean percentage invasion of 0.41 and 0.32 was recorded due to the inhibitory effect of BURK24 and BURK37 respectively (Figure 9).

### Effect on Pathogen-Induced Apoptosis

**Figure 10:**
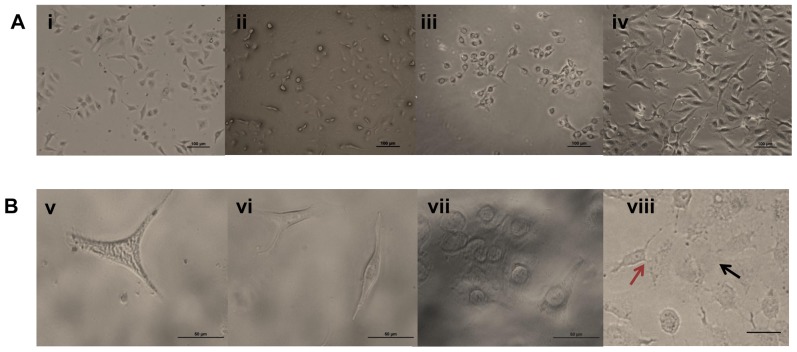
Morphological study on effect of mAbs on pathogen induced apoptosis using A549 cells. Phase contrast image of A549 cells in (**A**) 10× and (**B**) 40× magnification. (**i and v**) Phase contrast image of uninfected A549 control cells showing intact cell morphology. (**ii and vi**) Phase contrast image of A549 cells treated with *B. pseudomallei* NCTC 10274 for a dutration of 24 h in presence of BURK24. (**iii and vii**) A549 cells treated with *B. pseudomallei* NCTC 10274 in presence of BURK37. (**iv and viii**) A549 cells infected with *B. pseudomallei* NCTC 10274 showing apoptotic morphologies including reduction in cell volume and rupturing of cell membrane upon 24 h post-infection. (**viii**) Red arrow shows cell fusion and black arrow shows rupturing of cell membrane.

**Figure 11:**
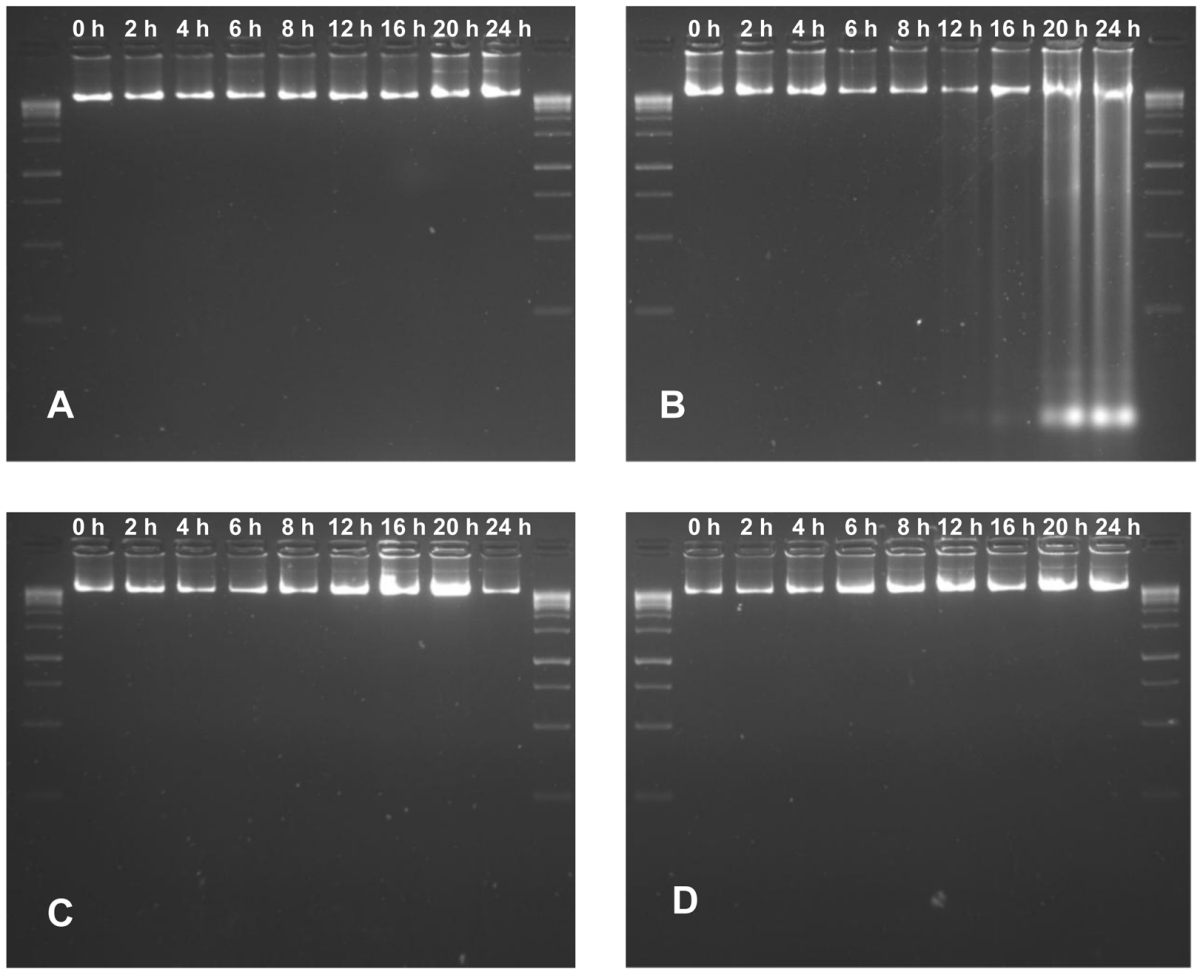
DNA damage assay of A549 cells. DNA damage assay was performed to study the pathogen induced apoptosis, where A549 cells were infected with *B. pseudomallei* NCTC 10274 in presence and absence of bacteriostatic concentration of BURK24 and BURK37. In a duration of 24 h, A549 cells were permeabilized and DNA was extracted at intervals of 0 h, 2 h, 4 h, 6 h, 8 h, 12 h, 16 h, 20 h and 24 h (**A**) DNA of A549 cells uninfected with *B. pseudomallei* (**B**) DNA of A549 cells infected with *B. pseudomallei*. DNA damage is noticed after 16 h of infection. (**C**) and (**D**) DNA of A549 cells infected with *B. pseudomallei* in presence of 30 µg/ml of BURK24 and 62.5 µg/ml of BURK37 respectively. The intactness of DNA in case of A549 cells treated with the pathogen in presence of mAbs is comparable to that of cells untreated with bacteria, inferring the efficiency of BURK24 and BURK37 to prevent apoptosis.

We extended our studies on the functional dimension of BURK24 and BURK37 by examining their ability to nullify the pathogen-induced apoptosis in mammalian cell lines. A comparative study was performed to observe the protection offered by BURK24 and BURK37 to A549 cells in presence of *B. pseudomallei* NCTC 10274 for duration of 24 h. A549 cells were infected with the bacterium to induce apoptosis and was referred as positive control. Whereas, A549 cells alone grown under defined normal condition in absence of the pathogen was considered as negative control. The study revealed that *B. pseudomallei* NCTC 10274 adequately induced apoptosis in A549 cells resulting in typical apoptotic morphological changes including decrease in cell volume, rupture of cell membrane and cell-to-cell fusion. On the other hand, no typical apoptotic morphological changes were observed in A549 cells when treated with the pathogen in presence of BURK24 and BURK37 separately was compared to negative control. The cell layer and volume appeared to be integrated. No cell fusions were observed (Figure 10). Complementary result was obtained in DNA fragmentation assay. Approximately 100 ng of DNA from tests, positive and negative control cells collected at each interval of the experiment was electrophoresed and stained with ethidium bromide for visualization under UV light (Figure 11). DNA fragmentation of A549 cells infected with *B. pseudomallei* NCTC 10274 was observed from 16 h of infection, suggesting the appreciable induction of apoptosis by the pathogen. However, DNA of A549 cells infected with *B. pseudomallei* NCTC 10274 in presence of bacteriostatic concentration of BURK24 and BURK37 were intact in agreement with that of the control cells throughout the experiment duration of 24 h. The result indicated that both the mAbs were protective for A549 cells, by preventing the pathogen to induce apoptosis.

## Discussion

In the present study, we focused on unraveling the functional attributes of monoclonal antibodies as anti-*B. pseudomallei* molecules. To achieve this, we adopted *in vitro* based approach on basis of four reasons. Firstly, *in vitro* experiments negate the possible effect of unknown factors on experiments which usually accompanies *in vivo* studies. Secondly, *in vitro* analysis more directly assess the authenticity of the work in terms of reproducibility. Thirdly, they are beneficial in terms of ethical considerations. Lastly, *in vitro*-based approach serves best to reduce expenses during preliminary studies as any molecule needs to be evaluated for its protective efficacy by several repetitive experiments.

Selection of an antigen was the primary challenge to generate promising mAbs with possible efficiency of establishing a passive immunity in the susceptible host. Inherent resistance to antibiotics, ability to form biofilm, invasiveness into both pathogocytic and non-phagocytic host cells are addressed for the strengthening pathogenesis of the bacterium. Outer membrane proteins (OMPs) that account for approximately 50% of the outer membrane mass in Gram-negative bacteria [42] seemed to be pertinent in this context. They promote serum resistance to the pathogen [42], [43], [44], [45], [46]. They play important role in adhesion [47] and invasion [48] of the bacterium to the host cells. Multi-drug efflux systems which confer multi-drug resistance to the pathogen also represent the OMPs [49]. In totality, OMPs play a key role in the Gram negative bacterial pathogenesis as they favor the pathogen to adapt in host niches [50]. Accordingly, we selected OMPs as the antigen for generation of potential antibodies, resulting in development of an array of mouse mAbs against crude outer membrane proteins extract of *B. pseudomallei* NCTC 10274 and screened for highly reactivity and promising mAbs. This resulted in encountering of two mAbs, namely BURK24 and BURK37, which were found to possess 100% reactivity for a total of eleven *B. pseudomallei* strains tested. They also exhibited reactivity with *B. cepacia*, a species of *Burkholderia* with clinical importance. BURK24 and BURK37 were deciphered to belong to IgG1 and IgM class of immunoglobulins, respectively. Additionally, Western blot analysis revealed that the epitope for BURK24 was the outer membrane proteins antigen present in multiple copies. The epitope for BURK37 was identified as LPS contaminants of the crude OMPs preparation. It is also well proven that LPS-specific antibodies majorly belong to IgM class of antibodies [51]. The isotyping and Western blot results shed light on two facts. Firstly, BURK24 and BURK37 belong to those immunoglobulin subclasses, which have been proven to have protective efficacy. To elaborate, IgG1, the immunoglobulin with highest biological half-life (21 days) is essayed to possess the activity of pathogen neutralization in tissues. IgM plays a major role in complement activation. Secondly, the epitope for BURK24 is undoubtedly present in multiple copies and that of BURK37 i.e., LPS is one of the major components of bacterial cell membrane. Thus we contemplated that BURK24 and BURK37 might possibly bind the pathogen efficiently throughout the surface and elicit changes in epitope or induce osmotic lysis resulting in the neutralization of pathogen activity and thus in turn demonstrate antimicrobial activity. Also, binding of mAb on the bacterial surface triggers the complement fixation, antibody dependent cell mediated cytotoxicity and opsonic activities on mAb-bacteria complex resulting in much more effective elimination of infection. To confirm this, we advanced to locate the binding region of both the mAbs on *B. pseudomallei* NCTC 10274 by designing a novel indirect immunofluorescence assay. We inferred a valuable functional property of BURK24 and BURK37 that they efficiently bind onto the surface of live bacteria adhering onto A549 cells resulting in fluorescence. This demonstrated the possible ability of both mAbs to reciprocate the same *in vivo* in infected hosts.

With this background, we examined BURK24 and BURK37 for their functional attributes as protective molecules in four different anti-*Burkholderia pseudomallei* activities *in vitro*. (1) As antimicrobial agents (2) As inhibitors of biofilm formation (3) As interrupting molecules in invasion of the pathogen into host cell (4) As agents to prevent bacterial-induced apoptosis.

When we channelized our work towards assessing the antimicrobial properties of BURK24 and BURK37, we selected the approved standards of CLSI which provides standards and guidelines to determine the *in vitro* susceptibility of antimicrobial molecules. To the best of our knowledge, this is the first report of applying CLSI standards for evaluating the antimicrobial activity of a monoclonal antibody. Use of monoclonal antibodies as bactericidal and bacteriostatic agent is justified, since both the antimicrobial actions are fundamental mechanisms of a host defence system. Accordingly, a successful “antibody susceptibility testing” was carried out to determine MIC and MBC of BURK24 and BURK37. The resulting MBC∶MIC ratio revealed that both the mAbs possessed bacteriostatic action on *B. pseudomallei*, since bacteriostatic activity of any antimicrobial has been defined as a ratio of MBC to MIC of >4. This provides valuable information on the potential antimicrobial action of both the mAbs *in vitro*. Kinetic study of bacteriostasis and time-kill curve analysis revealed that the antimicrobial activity of BURK24 and BURK37 was both concentration and time-dependent. This is because, a gradual decrease of 3 log_10_ in initial bacterial load was observed after fourth hour of the experiment.

Resistance of the biofilm forming *B. pseudomallei* to antimicrobial agents has been markedly proved [52], . This is because biofilm formation aids the bacteria to shield themselves in a polysaccharide matrix [54] and become inaccessible to the antibiotics. Thus biofilm formation results in the relapse of infection inspite of proper diagnosis and treatment. Nullifying the formation of biofilm has a remarkable importance in case of *B. pseudomallei* infections. As an attempt, BURK24 and BURK37 were studied for their efficacy in inhibiting the biofilm formation. Evaluation of biofilm formation of the strains in present study: *B. pseudomallei* NCTC 10274 and the *B. pseudomallei* strain 621 revealed that they possess variable capacity to form biofilm. *B. pseudomallei* NCTC 10274 was determined as a highly positive biofilm former whereas *B. pseudomallei* strain 621 was a low-grade biofilm former. However, BURK24 and BURK37 both showed significant inhibitory effect on biofilm formation of *B. pseudomallei* irrespective of the rate of biofilm formation of the pathogen. Biofilm formation depends on the successful establishment of interactions, between cell-to-cell and also cell-to-surface, which are regulated by various cell surface factors [55]. The inhibition of biofilm formation also indirectly suggests that the antigens of BURK24 and BURK37 i.e., OMPs and LPS might be involved in the biofilm formation of *B. pseudomallei*.

Currently, very sparse information is available about the mechanism of modulation induced in host cells by the invasive bacterium - *B. pseudomallei*. The bacterium has been reported to invade both phagocytic and non-phagocytic cells [56]. Adherence of the pathogen on the host cell surface is the primary step in establishment of pathogenesis of majority of infectious diseases. Similarly, as the first step of invasion, *B. pseudomallei* adheres onto the host cell membrane and further gets internalized [11]. Prevention of *B. pseudomallei* invasion into the host cells, thus promisingly prevents the pathogenesis. Efficiency of mAbs of this study was evaluated for the same. Prior to performing invasion inhibition assay, antibiotic resistance profile of both the strains, used in this study, was determined by antibiotic disc diffusion method. Among the antibiotics tested, chloramphenicol (30 µg/ml), ciprofloxacin (5 µg/ml) and tetracycline (30 µg/ml) inhibited the growth of the strains to a considerable degree (data not shown). Similar results were observed by Susan Shahin and David Proll in their study on adhesion and invasion of A549 cells by *B. pseudomallei* [57]. Accordingly, tetracycline was selected at a concentration of 150 µg/ml to kill extracellular bacterial cells and the killing activity at the mentioned concentration was confirmed for reproducibility (data not shown). The apparent inhibition of invasion of *B. pseudomallei* into A549 cells, represents a prototypical mode of action of BURK24 and BURK37 on prevention of invasiveness of the pathogen.

Induction of apoptosis by *B. pseudomallei* in epithelial cell lines has been well studied [14], [58]. Similar results were obtained in the present study, where *B. pseudomallei* NCTC 10274 was found to cause morphological changes in A549 epithelial cells; including reduction in cell volume, cell fusion and rupture of cell membrane. Concurrent DNA damage analysis inferred that the strain could induce DNA damage after 16 h of infection, as demonstrated by different strains of the pathogen in earlier studies [14]. On the other hand, the morphological changes in A549 cells infected with *B. pseudomallei* NCTC 10274 in presence of BURK24 and BURK37 appeared to be intermediate between control cells and those infected with bacteria alone throughout the experimental duration. They appeared to be under physiological stress. We predict the reason for stress could be the establishment of pathogenesis by a few intracellular bacterial cells (0.41% and 0.32% of infected bacteria in presence of BURK24 and BURK37 respectively, as determined in invasion inhibition assay presented in this study) in the later stage of infection. However, DNA of the epithelial cells remained intact throughout the experiment, revealing the potential of both the mAbs in preventing *B. pseudomallei* to induce apoptosis in host cells and confer considerable protection.

The results derived in all the *in vitro* experiments in the current study provides an insight into a possible protective intervention against *B. pseudomallei* infections – Passive immunization of the susceptible host with the mAbs possessing antibacterial, time-dependent bacteriostatic, anti-biofilm, anti-invasive and anti-apoptotic attributes i.e., BURK24 and BURK37 possibly provides a complete cure from *B. pseudomallei* infections. The additional reactivity of the mAbs with *B. cepacia* also projects the possibility of obtaining cross protection against *B. cepacia* infections – one of the major challenges in case of cystic fibrosis. This needs additional and intense clinical validation of the results by performing *in vivo* experiments in murine models by employing mouse, scFv-based and humanized BURK24 and BURK37.

## References

[1] White NJ (2003) Melioidosis. Lancet 361 9370: 1715–1722. 12767750 10.1016/s0140-6736(03)13374-0

[2] Currie BJ, Ward L, Cheng AC (2010) The epidemiology and clinical spectrum of melioidosis: 540 cases from the 20 year Darwin prospective study. PLoS Negl Trop Dis 4 11: e900 10.1371/journal.pntd.0000900 21152057 PMC2994918

[3] Chetchotisakd P, Chierakul W, Chaowagul W, Anunnatsiri S, Phimda K, et al (2013) Trimethoprim-sulfamethoxazole versus trimethoprim-sulfamethoxazole plus doxycycline as oral eradicative treatment for melioidosis (MERTH): a multicenter, double-blind, non-inferiority, randomized controlled trial. Lancet 10.1016/S0140-6736(13)61951-0 PMC3939931 24284287

[4] Chaowagul W, Suputtamongkol Y, Dance DA, Rajchanuvong A, Pattara-arechachai J, et al (1993) Relapse in melioidosis: incidence and risk factors. J Infect Dis 168 5: 1181–1185 10.1093/infdis/168.5.1181 8228352

[5] Behera B, Prasad Babu TL, Kamalesh A, Reddy G (2012) Ceftazidime resistance in *Burkholderia pseudomallei*: First report from India. Asian Pac J Trop Med 5 4: 329–330. 22449529 10.1016/S1995-7645(12)60050-9

[6] Thibault FM, Hernandez E, Vidal DR, Girardet M, Cavallo JD (2004) Antibiotic susceptibility of 65 isolates of *Burkholderia pseudomallei* and *Burkholderia mallei* to 35 antimicrobial agents. J Antimicrob Chemother 54 6: 1134–1138. 15509614 10.1093/jac/dkh471

[7] Jenney AW, Lum G, Fisher DA, Currie BJ (2001) Antibiotic susceptibility of *Burkholderia pseudomallei* from tropical northern Australia and implications for therapy of melioidosis. Int J Antimicrob Agents 17 2: 109–113. 11165114 10.1016/s0924-8579(00)00334-4

[8] Dance DA, Wuthiekanun V, Chaowagul W, White NJ (1989) The antimicrobial susceptibility of *Pseudomonas pseudomallei*. Emergence of resistance *in vitro* and during treatment. J Antimicrob Chemother 24 3: 295–309. 2681116 10.1093/jac/24.3.295

[9] Brekke OH, Sandlie I (2003) Therapeutic antibodies for human diseases at the dawn of the twenty-first century. Nat Rev Drug Discov 2 1: 52–62. 12509759 10.1038/nrd984

[10] Ter Meulen J (2007) Monoclonal antibodies for prophylaxis and therapy of infectious diseases. Expert Opin Emerg Drugs 12 4: 525–540. 17979597 10.1517/14728214.12.4.525

[11] Jones AL, Beveridge TJ, Woods DE (1996) Intracellular survival of *Burkholderia pseudomallei* . Infect Immun 64 3: 782–790. 8641782 10.1128/iai.64.3.782-790.1996 PMC173838

[12] Kespichayawattana W, Rattanachetkul S, Wanun T, Utaisincharoen P, Sirisinha S (2000) *Burkholderia pseudomallei* induces cell fusion and actin-associated membrane protrusion: a possible mechanism for cell- to- cell spreading. Infect Immun 68 9: 5377–5384. 10948167 10.1128/iai.68.9.5377-5384.2000 PMC101801

[13] Suparak S, Kespichayawattana W, Haque A, Easton A, Damnin S, et al (2005) Multinucleated giant cell formation and apoptosis in infected host cells is mediated by *Burkholderia pseudomallei* type III secretion protein BipB. J Bacteriol 187 18: 6556–6560. 16159789 10.1128/JB.187.18.6556-6560.2005 PMC1236626

[14] Koh SF, Tay ST, Puthucheary SD (2013) Colonial morphotypes and biofilm forming ability of *Burkholderia pseudomallei* . Trop Biomed 30 3: 428–433. 24189672

[15] Sawasdidoln C, Taweechaisupapong S, Sermswan RW, Tattawasart U, Tungpradabkul S, et al (2010) Growing *Burkholderia pseudomallei* in biofilm stimulating conditions significantly induces antimicrobial resistance. PLoS ONE 5 2: e9196 10.1371/journal.pone.0009196 20169199 PMC2820546

[16] Finke M, Muth G, Reichhelm T, Thoma M, Duchene M, et al (1991) Protection of immunosuppressed mice against infection with *Pseudomonas aeruginosa* by recombinant *P. aeruginosa* lipoprotein I and lipoprotein I-specific monoclonal antibodies. Infect Immun 59 4: 1251–1254. 1706316 10.1128/iai.59.4.1251-1254.1991 PMC257835

[17] Toropainen M, Saarinen L, Vidarsson G, Kayhty H (2006) Protection by meningococcal outer membrane protein PorA-specific antibodies and a serogroup B capsular polysaccharide-specific antibody in complement-sufficient and C6-deficient infant rats. Infect Immun 74 5: 2803–2808. 16622217 10.1128/IAI.74.5.2803-2808.2006 PMC1459742

[18] Di Padova FE, Brade H, Barclay GR, Poxton IR, Liehl E, et al (1993) A broadly cross-protective monoclonal antibody binding to *Escherichia coli* and *Salmonella* lipopolysaccharides. Infect Immun 61 9: 3863–3872. 8359907 10.1128/iai.61.9.3863-3872.1993 PMC281087

[19] Levitz SM, Farrell TP, Maziarz RT (1991) Killing of *Cryptococcus neoformans* by human peripheral blood mononuclear cells stimulated in culture. J Infect Dis 163 5: 1108–1113. 2019758 10.1093/infdis/163.5.1108

[20] Akiyama M, Oishi K, Tao M, Matsumoto K, Pollack M (2000) Antibacterial properties of *Pseudomonas aeruginosa* immunotype 1 lipopolysaccharide-specific monoclonal antibody (MAb) in a murine thigh infection model: Combined effects of MAb and ceftazidime. Microbiol Immunol 44 8: 629–635 10.1111/j.1348-0421.2000.tb02543.x 11021392

[21] Silva EB, Dow SW (2013) Development of *Burkholderia mallei* and *pseudomallei* vaccines. Front Cell Infect Microbiol 3: 11 10.3389/fcimb.2013.00010 23508691 PMC3598006

[22] Jones SM, Ellis JF, Russell P, Griffin KF, Oyston PC (2002) Passive protection against *Burkholderia pseudomallei* infection in mice by monoclonal antibodies against capsular polysaccharide, lipopolysaccharide or proteins. J Med Microbiol 51 12: 1055–1062. 12466403 10.1099/0022-1317-51-12-1055

[23] AuCoin DP, Reed DE, Marlenee NL, Bowen RA, Thorkildson P, et al (2012) Polysaccharide specific monoclonal antibodies provide passive protection against intranasal challenge with *Burkholderia pseudomallei* . PLoS ONE 7 4: e35386 10.1371/journal.pone.0035386 22530013 PMC3328442

[24] Bottex C, Gauthier YP, Hagen RM, Finke EJ, Splettstosser WD, et al (2005) Attempted passive prophylaxis with a monoclonal anti-*Burkholderia pseudomallei* exopolysaccharide antibody in a murine model of melioidosis. Immunopharmacol Immunotoxicol 27 4: 565–583. 16435577 10.1080/08923970500493995

[25] Nelson M, Prior JL, Lever MS, Jones HE, Atkins TP, Titball RW (2004) Evaluation of lipopolysaccharide and capsular polysaccharide as subunit vaccines against experimental melioidosis. J Med Microbiol 53 12: 1177–1182. 15585494 10.1099/jmm.0.45766-0

[26] Kulkarni RD, Jain P, Ajantha GS, Shetty J, Chunchanur S, et al (2010) Fatal *Burkholderia pseudomallei* septicaemia in a patient with diabetes. Indian J Med Res 131: 584–585. 20424312

[27] Brook MD, Currie B, Desmarchelier PM (1997) Isolation and identification of *Burkholderia pseudomallei* from soil using selective culture techniques and the polymerase chain reaction. J Appl Microbiol 82 5: 589–596. 9172400

[28] Crosa JH, Hodges LL (1981) Outer membrane proteins induced under conditions of iron limitation in the marine fish pathogen *Vibrio anguillarum* . Infect Immun 31 1: 223–227. 7194320 10.1128/iai.31.1.223-227.1981 PMC351773

[29] Lowry OH, Rosebrough NJ, Farr AL, Randall RJ (1951) Protein measurement with the Folin phenol reagent. J Biol Chem 193 1: 265–275. 14907713

[30] Laemmli UK (1970) Cleavage of structural proteins during the assembly of the head of bacteriophage T4. Nature 227: 680–685. 5432063 10.1038/227680a0

[31] Kohler G, Milstein C (1975) Continuous cultures of fused cells secreting antibody of redefined specificity. Nature 256: 495–497. 1172191 10.1038/256495a0

[32] Burnette WN (1981) “Western blotting”: electrophoretic transfer of proteins from sodium dodecyl sulfate–polyacrylamide gels to unmodified nitrocellulose and radiographic detection with antibody and radioiodinated protein A. Anal Biochem 112 2: 195–203. 6266278 10.1016/0003-2697(81)90281-5

[33] Westerman RB, He Y, Keen JE, Littledike ET, Kwang J (1997) Production and charecterisation of monoclonal antibodies specific for the lipopolysaccharide of *Escherichia coli* 0157. J Clin Microbiol 35 3: 679–684. 9041412 10.1128/jcm.35.3.679-684.1997 PMC229650

[34] NCCLS (1999) Methods for determining bactericidal activity of antibacterial agents; approved guideline. NCCLS document M26-A. Villanova, PA: NCCLS.

[35] NCCLS (2006) Methods for dilution antimicrobial susceptibility tests for bacteria that grow aerobically; Approved Standard. Clinical and Laboratory Standards Institute document M7-A7 [ISBN 1-56238-587-9] Pennsylvania: Clinical and Laboratory Standards Institute.

[36] Sun D, Accavitti MA, Bryers JD (2005) Inhibition of biofilm formation by monoclonal antibodies against *Staphylococcus epidermidis* RP62A accumulation- associated protein. Clin Diagn Lab Immunol 12 1: 93–100. 15642991 10.1128/CDLI.12.1.93-100.2005 PMC540198

[37] Knobloch JK, Horstkotte MA, Rohde H, Kaulfers PM, Mack D (2002) Alcoholic ingredients in skin disinfectants increase biofilm expression of *Staphylococcus epidermidis* . J Antimicrob Chemother 49 4: 683–687. 11909845 10.1093/jac/49.4.683

[38] Fulop MJ, Webber T, Manchee RJ, Kelly DC (1991) Production and characterization of monoclonal antibodies directed against the lipopolysaccharide of *Francisella tularensis* . J Clin Microbiol 29 7: 1407–1412. 1885735 10.1128/jcm.29.7.1407-1412.1991 PMC270126

[39] Griep RA, van Twisk C, van Beckhoven JR, van der Wolf JM, Schots A (1998) Development of specific recombinant monoclonal antibodies against the lipopolysaccharide of *Ralstonia solanacearum* race 3. Phytopathology 88 8: 795–803. 18944885 10.1094/PHYTO.1998.88.8.795

[40] Mitov I, Georgiev G, Ivanova R, Petrov D, Haralambieva I, et al (2003) Monoclonal antibody against O:5 Salmonella antigen cross-reacts with unidentified lipopolysaccharide epitope of *Salmonella* serogroup O:8 (C_2_-C_3_). FEMS Microbiol Lett 225: 299–304. 12951256 10.1016/S0378-1097(03)00533-0

[41] Anuntagool N, Wuthiekanun V, White NJ, Currie BJ, Sermswan RW, et al (2006) Lipopolysaccharide heterogeneity among Burkholderia pseudomallei from different geographic and clinical origins. Am J Trop Med Hyg 74 3: 348–352. 16525090

[42] Koebnik R, Locher KP, Van Gelder P (2000) Structure and function of bacterial outer membrane proteins: barrels in a nutshell. Mol Microbiol 37 2: 239–253. 10931321 10.1046/j.1365-2958.2000.01983.x

[43] Ram S, McQuillen DP, Gulati S, Elkins C, Pangburn MK, et al (1998) Binding of complement factor H to loop 5 of porin protein 1A: a molecular mechanism of serum resistance of nonsialylated *Neisseria gonorrhoeae* . J Exp Med 188 4: 671–680. 9705949 10.1084/jem.188.4.671 PMC2213355

[44] Morello JA, Bohnhoff M (1989) Serovars and serum resistance of *Neisseria gonorrhoeae* from disseminated and uncomplicated infections. J Infect Dis 160 6: 1012–1017. 2511251 10.1093/infdis/160.6.1012

[45] Hellwage J, Meri T, Heikkila T, Alitalo A, Panelius J, et al (2001) The complement regulator factor H binds to the surface protein OspE of *Borrelia burgdorferi* . J Biol Chem 276 11: 8427–8435. 11113124 10.1074/jbc.M007994200

[46] Weiser JN, Gotschlich EC (1991) Outer membrane protein A (OmpA) contributes to serum resistance and pathogenicity of *Escherichia coli* K-1. Infect Immun 59 7: 2252–2258. 1646768 10.1128/iai.59.7.2252-2258.1991 PMC258003

[47] Heesemann J, Gruter L (1987) Genetic evidence that the outer membrane protein YOP1 of *Yersinia enterocolitica* mediates adherence and phagocytosis resistance to human epithelial cells. FEMS Microbiol Lett 40 1: 37–41.

[48] Kim K, Kim KP, Choi J, Lim JA, Lee J, et al (2010) Outer membrane proteins A (OmpA) and X (OmpX) are essential for basolateral invasion of *Cronobacter sakazakii* . Appl Environ Microbiol 76 15: 5188–5198. 20543055 10.1128/AEM.02498-09 PMC2916488

[49] Masuda N, Sakagawa E, Ohya S (1995) Outer membrane proteins responsible for multi drug resistance in *Pseudomonas aeruginosa* . Antimicrob Agents Chemother 39 3: 645–649. 7793866 10.1128/AAC.39.3.645 PMC162598

[50] Lin J, Huang S, Zhang Q (2002) Outer membrane proteins: key players for bacterial adaptation in host niches. Microbes Infect 4 4: 325–331. 11909743 10.1016/s1286-4579(02)01545-9

[51] Pollack M, Koles NL, Preston MJ, Brown BJ, Pier GB (1995) Functional properties of isotype-switched immunoglobulin M (IgM) and IgG monoclonal antibodies to *Pseudomonas aeruginosa* lipopolysaccharide. Infect Immun 63 11: 4481–4488. 7591089 10.1128/iai.63.11.4481-4488.1995 PMC173638

[52] Sawasdidoln C, Taweechaisupapong S, Sermswan RW, Tattawasart U, Tungpradabkul S, et al (2010) Growing *Burkholderia pseudomallei* in biofilm stimulating conditions significantly induces antimicrobial resistance. PLoS ONE 5 2: e9196 10.1371/journal.pone.0009196 20169199 PMC2820546

[53] Pibalpakdee P, Wongratanacheewin S, Taweechaisupapong S, Niumsup PR (2012) Diffusion and activity of antibiotics against *Burkholderia pseudomallei* biofilms. Int J Antimicrob Agents 39 4: 356–359 10.1016/j.ijantimicag.2011.12.010 22364716

[54] Dunne WM Jr (2002) Bacterial adhesion: Seen any good biofilm lately? Clin Microbiol Rev 15 2: 155–166. 11932228 10.1128/CMR.15.2.155-166.2002 PMC118072

[55] Bogino PC, Oliva Mde L, Sorroche FG, Giordano W (2013) The role of bacterial biofilms and surface components in plant-bacterial associations. Int J Mol Sci 14 8: 15838–15859. 23903045 10.3390/ijms140815838 PMC3759889

[56] Allwood EM, Devenish RJ, Prescott M, Adler B, Boyce JD (2011) Strategies for intracellular survival of *Burkholderia pseudomallei* . Front Microbiol 2: 170 10.3389/fmicb.2011.00170 22007185 PMC3159172

[57] Susan Shahin, David Proll (2004) Adhesion and invasion of human lung epithelial cells by *Burkholderia pseudomallei* DSTO-TR-1584. Available: http://www.dsto.defence.gov.au/publications/3435/DSTO-TR-1584.pdf

[58] Phewkliang A, Wongratanacheewin A, Chareonsudjai S (2010) Role of *Burkholderia pseudomallei* in the invasion, replication and induction of apoptosis in human epithelial cell lines. Southeast Asian J Trop Med Public Health 41 5: 1164–1176. 21073038

